# Neutrophil-derived migrasomes are an essential part of the coagulation system

**DOI:** 10.1038/s41556-024-01440-9

**Published:** 2024-07-12

**Authors:** Dong Jiang, Lin Jiao, Qing Li, Renxiang Xie, Haohao Jia, ShiHui Wang, Yining Chen, Siyuan Liu, Dandan Huang, Jiajia Zheng, Wenhao Song, Ying Li, JianFeng Chen, Jinsong Li, Binwu Ying, Li Yu

**Affiliations:** 1 State Key Laboratory of Membrane Biology, Tsinghua University–Peking University Joint Center for Life Sciences, Beijing Frontier Research Center for Biological Structure, School of Life Sciences, Tsinghua University, Beijing, China; 2 Department of Laboratory Medicine, West China Hospital, Sichuan University, Chengdu, China; 3 Shanghai Key Laboratory of Molecular Andrology, Shanghai Institute of Biochemistry and Cell Biology, Center for Excellence in Molecular Cell Science, Chinese Academy of Sciences, Shanghai, China; 4 State Key Laboratory of Multi-Cell Systems, Shanghai Institute of Biochemistry and Cell Biology, Center for Excellence in Molecular Cell Science, Chinese Academy of Sciences, Shanghai, China; 5 Department of Laboratory Medicine, Peking University Third Hospital, Beijing, People’s Republic of China

**Keywords:** Organelles, Circulation

## Abstract

Migrasomes are organelles that are generated by migrating cells. Here we report the key role of neutrophil-derived migrasomes in haemostasis. We found that a large number of neutrophil-derived migrasomes exist in the blood of mice and humans. Compared with neutrophil cell bodies and platelets, these migrasomes adsorb and enrich coagulation factors on the surface. Moreover, they are highly enriched with adhesion molecules, which enable them to preferentially accumulate at sites of injury, where they trigger platelet activation and clot formation. Depletion of neutrophils, or genetic reduction of the number of these migrasomes, significantly decreases platelet plug formation and impairs coagulation. These defects can be rescued by intravenous injection of purified neutrophil-derived migrasomes. Our study reveals neutrophil-derived migrasomes as a previously unrecognized essential component of the haemostasis system, which may shed light on the cause of various coagulation disorders and open therapeutic possibilities.

## Abstract (web summary)

Jiang et al. document an abundance of neutrophil-derived migrasomes in the blood of mice and humans and show that migrasomes are enriched in coagulation factors, accumulate at sites of injury and trigger platelet activation and clot formation.

## Main

A complicated, interrelated system referred to as haemostasis maintains the fluidity of blood while allowing rapid repair of injured blood vessels^1^. The components of the haemostasis system include platelets, blood vessel walls and coagulation factors^2^. Blood vessel injury exposes the subendothelial matrix, which initiates platelet adhesion by binding to various receptors on the surface of platelets. This subsequently triggers the activation and aggregation of platelets, resulting in the formation of a platelet plug. At the same time, activation of the coagulation cascade leads to the generation of thrombin, which cleaves fibrinogen to insoluble fibrin. Fibrin forms a crosslinked mesh, which greatly strengthens the platelet plug and produces a haemostatic plug to stop the bleeding^1,3,4^. Besides these well-established components, it is unknown whether or not there are other essential components of the haemostasis system.

Migrasomes are organelles of migrating cells. During cell migration, long membrane tethers named retraction fibres are left behind the trailing edge of cells, and large vesicular structures named migrasomes grow from the retraction fibres^5^. Migrasomes are released from cells when the cells move away. The formation of migrasomes is driven by the assembly of tetraspanin-enriched macrodomains^6^; thus, molecules associated with tetraspanin-enriched microdomains, such as integrins, are highly enriched in migrasomes^7^. It has been reported that knockdown or knockout of migrasome-promoting tetraspanins impairs migrasome formation^6,8,9^. The formation of migrasomes has been observed in various in vivo settings, and migrasomes have been shown to play important roles in zebrafish organogenesis (by releasing signalling molecules^8^), mitochondrial quality control (by shedding damaged mitochondria^9^) and the modulation of surrounding cells (by lateral transfer of messenger RNA^10^).

## Results

### Neutrophils generate a large number of migrasomes in blood

**Fig. 1:**
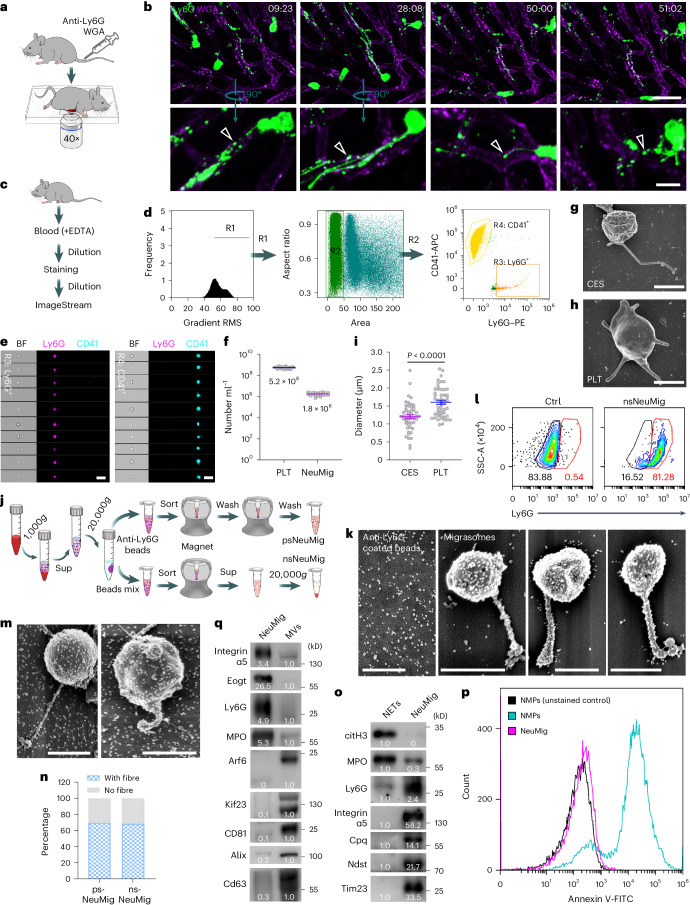
Circulating neutrophils generate a large number of migrasomes in blood vessels. **a**, Diagram of the procedure for intravital imaging of mouse liver. **b**, Intravital imaging of neutrophils in mouse liver. Neutrophils were labelled with PE–anti-Ly6G (Gr1; green) and blood vessels were labelled with AF647–WGA (purple). Scale bars, 30 μm (top) and 10 μm (magnified; bottom). The arrowheads indicate migrasomes. **c**, Diagram of the procedure for preparing samples for imaging flow cytometry analysis. **d**, Gating strategy of imaging flow cytometry analysis. Blood was stained with PE–anti-Ly6G and APC–anti-CD41. Neutrophil-derived migrasomes (R3; Ly6G^+^) and platelets (R4; CD41^+^) were gated from the small particle population (R2). **e**, Images of neutrophil-derived migrasomes and platelets from ImageStream. Scale bars, 10 μm. **f**, Quantification of neutrophil-derived migrasomes (NeuMigs) and platelets (PLTs) in mouse blood by ImageStream analysis. *n* = 20 mice. **g**,**h**, SEM images of a CES (**g**) and a platelet (**h**). Scale bars, 1 μm. **i**, Measurement of the diameter of migrasomes and platelets. *n* = 50 particles per group. **j**, Diagram of the procedure for positive (top) and negative (bottom) isolation of neutrophil-derived migrasomes from blood. **k**, SEM images of anti-Ly6G-conjugated beads and positively sorted neutrophil-derived migrasomes (psNeuMigs). Scale bars, 1 μm. **l**, Flow cytometry analysis of negatively sorted neutrophil-derived migrasomes (nsNeuMigs) stained with PE–anti-Ly6G. Particles positively isolated with the kit beads served as a control (Ctrl). Samples from 20 mice were pooled and analysed together. **m**, SEM images of nsNeuMig incubated with anti-Ly6G-conjugated magnetic beads. Scale bars, 1 μm. **n**, Percentage of psNeuMig and nsNeuMig with or without retraction fibres. *n* = 43 for psNeuMig and *n* = 54 for nsNeuMig. **o**, nsNeuMigs and NETs were normalized with total protein and subjected to western blot analysis using antibodies against markers for NETs and migrasomes. **p**, Flow cytometry analysis of nsNeuMig and NMPs after staining with Annexin V-FITC. Unstained NMPs served as a negative control. **q**, Isolated neutrophil-derived migrasomes and microvesicles were normalized with total protein and subjected to western blot analysis using antibodies against the indicated molecules. All statistical data are presented as means ± s.e.m. *P* values were calculated using a two-tailed, unpaired *t*-test. The western blot grey values were quantified using ImageJ. Source numerical data and unprocessed blots are available in Source Data Fig. 1. RMS, root mean square. BF, bright field; Sup, supernatant. Source data

Previously, we reported that circulating neutrophils generate neutrophil-derived migrasomes in the circulation^9,11^, which we confirmed here by intravital labelling of neutrophils using Ly6G antibody (Fig. 1a,b and Supplementary Video 1). Using imaging flow cytometry analysis of mouse blood, we found that blood contains structures notably smaller than cells, including an Ly6G^+^ sub-population (Fig. 1c,d). Imaging analysis showed that these Ly6G^+^ structures are small vesicles about 1 μm in size, resembling in vivo neutrophil-derived migrasomes (Fig. 1e). As a positive control, we used anti-CD41 antibody to detect platelets, which also helped us compare the abundance of Ly6G^+^ vesicles (Fig. 1e). We found that the number of Ly6G^+^ vesicles is about 1/300 of the number of platelets (Fig. 1f). The number of Ly6G^+^ vesicles can be 1.8 × 10^6^ ml^−1^ blood.

**Extended Data Fig. 1:**
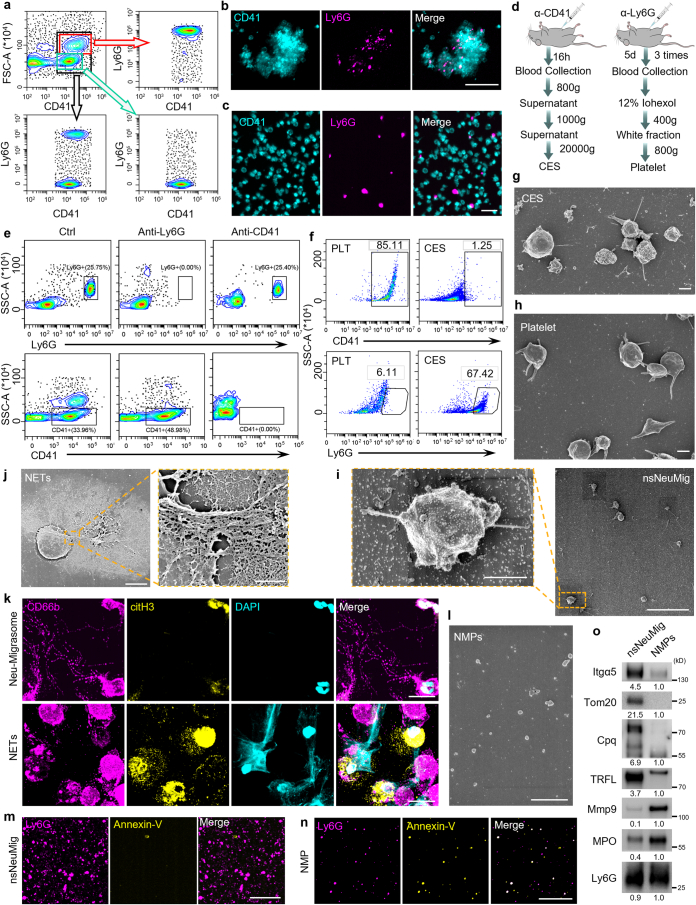
Isolation and characterization of neutrophil-derived migrasomes. **a**, Flow cytometry analysis of the blood white cells after staining with PE-anti-Ly6G and APC-anti-CD41 by CytoFLEX. **b**, The CD41^+^ Ly6G^+^ population of the white cells was sorted by MoFlo-Astrios-EQ and imaged by Dragonfly confocal microscopy. Scale bar, 20 μm. **c**, Platelets were isolated and stained with PE-anti-Ly6G and APC-anti-CD41 for Dragonfly imaging. Scale bar, 10 μm. **d**, Diagram of the procedures for preparing CES from the blood of platelet-depleted mice (left), and for purifying platelets from the blood of neutrophil-depleted mice (right). **e**, Flow cytometry analysis of whole blood cells from control (left panels), neutrophil-depleted (middle panels), and platelet-depleted mice (right panels) after staining with PE-anti-Ly6G or APC-anti-CD41. Samples from five mice were pooled and analyzed together. **f**, Flow cytometry analysis of purified platelets (PLTs) and CES after staining with PE-anti-Ly6G and APC-anti-CD41. Samples from five mice were pooled and analyzed together. **g-h**, SEM image of CESs (**g**) or platelets (**h**). These results come from the same experiments as Fig. 1g, h. Scale bar, 1 μm. **i**, SEM images of nsNeuMigs which were incubated with anti-Ly6G-conjugated magnetic beads. Scale bar, 10 μm. The migrasome in the dashed-box is enlarged at the left. Scale bar, 1 μm. **j**, SEM images of NETs. Scale bar, 5 μm (left); 1 μm (enlarged). **k**, Immunofluorescence staining images of migrasomes and NETs. Scale bar, 20 μm. **l**, SEM image of NMPs. Scale bar, 1 μm. **m-n**, Dragonfly confocal images of nsNeuMigs (**m**) or NMPs (**n**) stained with PE-anti-Ly6G (Gr1) and Annexin V-FITC. Scale bar, 20 μm. **o**. NMPs were compared with nsNeuMig by western blot analysis. The samples were normalized with total protein level. Gray values of western blots were quantified by Image J. Source unprocessed blots are available in source data. Source data

To isolate Ly6G^+^ vesicles from blood, we centrifuged it at 1,000*g* for 15 min to remove most platelets, then used fluorescence-activated cell sorting (FACS) with anti-CD41 antibody to check for pellets and stained with anti-Ly6G to assess Ly6G^+^ structure loss during centrifugation. Surprisingly, we detected a CD41^+^Ly6G^+^ population, revealed by confocal microscopy as aggregates of platelets and Ly6G^+^ vesicles (Extended Data Fig. 1a,b). This finding, considering their similar sizes, led us to check standard platelet collection protocols for contamination. We found that platelet-rich plasma from standard protocols contains Ly6G^+^ vesicles (Extended Data Fig. 1c).

Ly6G^+^ vesicles can aggregate with platelets, complicating their isolation. To address this, we depleted platelets or neutrophils in mice using anti-CD41 or anti-Ly6G antibodies, respectively, achieving depletion within 14 h for platelets and 5 d for neutrophils. We then isolated crude extracellular structures (CESs) from platelet-depleted mice by first using low-speed centrifugation to remove blood cells, followed by high-speed centrifugation, resulting in minimal platelet contamination and predominantly Ly6G^+^ CESs, indicating a neutrophil origin (Extended Data Fig. 1d–f). Similarly, we collected platelets from neutrophil-depleted mice. Scanning electron microscopy (SEM) on these isolated structures showed that neutrophil-derived migrasomes and platelets both exhibit round bodies with projections (Fig. 1g,h and Extended Data Fig. 1g,h), typical of migrasomes, with sizes of 1.2 µm for migrasomes and 1.6 µm for platelets (Fig. 1i). To further purify neutrophil-derived migrasomes from the crude preparation of extracellular structures described above, we carried out an immune isolation protocol. The CESs were incubated with anti-Ly6G-conjugated magnetic beads and then magnetic sorting was performed (Fig. 1j, top). SEM analysis showed that the resulting structures were neutrophil-derived migrasomes densely coated with magnetic beads (Fig. 1k). We also carried out immune purification using a negative selection kit for neutrophils (Fig. 1j, bottom). FACS analysis showed that the negative selection procedure resulted in a preparation in which more than 80% of extracellular structures were positive for Ly6G (Fig. 1l). For both the positively and negatively selected structures, SEM revealed that the majority had attached retraction fibres, which is the defining feature of migrasomes (Fig. 1k,m,n and Extended Data Fig. 1i). Neutrophils release neutrophil extracellular traps (NETs), so we used western blot analysis to check for NET contamination in our preparations, focusing on markers of NETs and migrasomes. We found that migrasome markers, including integrin α_5_, Cpq, Ndst and Ly6G, were enriched^7,9,11,12^, along with the mitochondrial marker Tim23, consistent with our findings of damaged mitochondria in neutrophil-derived migrasomes^9^ (Fig. 1o). In contrast, the NET marker citrullinated histone H3 was absent^13^. Further SEM and confocal microscopy confirmed that the structures we isolated were migrasomes, not NETs, as they lacked the dense mesh of fine filaments typical of NETs and their specific markers^14^ (Extended Data Fig. 1j,k). Collectively, these data suggest that our preparation was not contaminated with a notable number of NETs.

It is well established that neutrophils can generate neutrophil-derived microparticles (NMPs) or microvesicles. NMPs are small vesicles with a diameter of 0.05–1.00 μm originating directly from the plasma membrane^15–17^. To compare the NMPs and neutrophil-derived migrasomes, we purified NMPs following well-established protocols from the literature^16,18^. SEM imaging showed that NMPs are small vesicles with a diameter of ~100 nm and, notably, there is no fibre-like structure attached to NMPs (Extended Data Fig. 1l). Moreover, FACS analysis and confocal imaging showed that the vast majority of neutrophil-derived migrasomes we purified did not expose phosphatidylserine on the outer membrane leaflet (Fig. 1p and Extended Data Fig. 1m). Phosphatidylserine exposure on the outer leaflet is the defining feature of NMPs^15–17^, which we confirmed here (Fig. 1p and Extended Data Fig. 1n). Western blot analysis also revealed the difference between neutrophil-derived migrasomess and NMPs (Extended Data Fig. 1o). We also checked whether or not neutrophil-derived migrasomes isolated from blood are contaminated by microvesicles and exosomes. Our purified neutrophil-derived migrasomes did not enrich Arf6 and Kif23, the markers for microvesicles^19,20^, and they contained very little CD81, Alix and CD63, the markers for exosomes^21^ (Fig. 1q). Together, these observations indicate that the structures we purified from blood were not NETs or NMPs, but rather were neutrophil-derived migrasomes.

### Coagulation factors are enriched in neutrophil-derived migrasomes

**Fig. 2:**
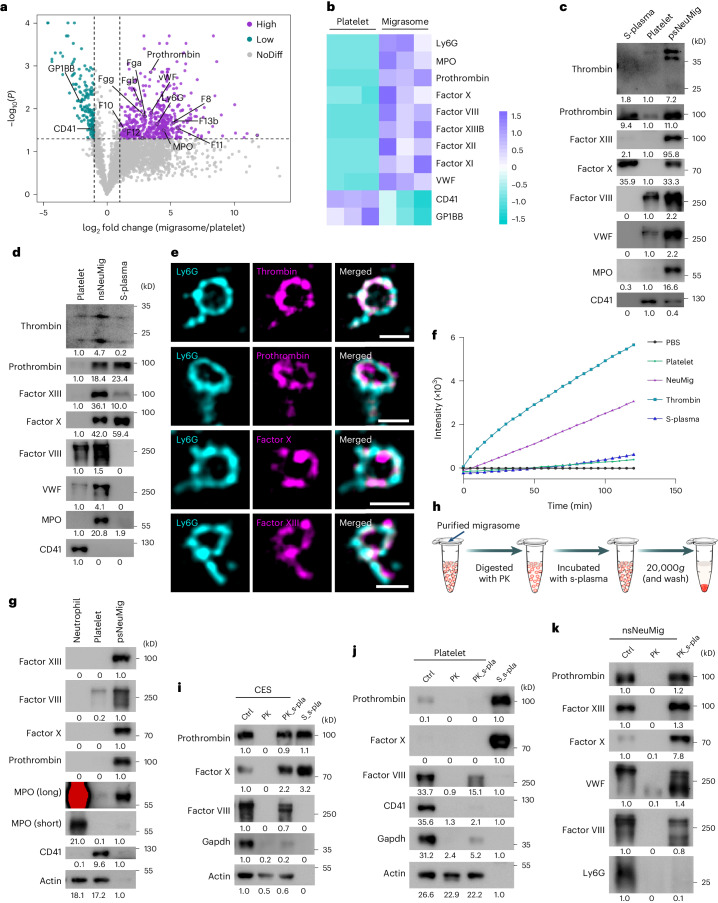
Coagulation factors are enriched in neutrophil-derived migrasomes. **a**, Volcano plot showing the differential abundance of proteins in isolated neutrophil-derived migrasomes versus platelets. Migrasomes and platelets were subjected to label-free quantitative mass spectrometry analysis. The purple dots represent a migrasome/platelet abundance ratio of ≥2 (*P* < 0.05) and the cyan dots represent a migrasome/platelet abundance ratio of ≤0.5 (*P* < 0.05). *n* = 3 biologically independent experiments. *P* values were calculated using a two-tailed, unpaired *t*-test. **b**, Heat map of the distribution of coagulation factors in platelets and neutrophil-derived migrasomes. The coloured scale bars represent relative values. **c**,**d**, psNeuMigs (**c**) or nsNeuMigs (**d**) were isolated from the blood of platelet-depleted mice and platelets were isolated from the blood of neutrophil-depleted mice. S-plasma is the supernatant after centrifuging plasma at 20,000*g*, 4 ℃ for 1 h. These samples were normalized with total protein and subjected to western blot analysis. **e**, Confocal microscopy images of purified neutrophil-derived migrasomes revealed by immunofluorescence staining using anti-Ly6G and anti-thrombin, anti-prothrombin, anti-factor X or anti-factor XIII. Scale bars, 1 μm. **f**, Thrombin activity assay using the internally quenched 5-FAM/QXL-520 FRET substrate of thrombin. nsNeuMigs, platelets and s-plasma were prepared and normalized with total protein, then mixed with the thrombin substrate for fluorescence detection by EnSpire microplate reader. Thrombin (0.5 U ml^−1^) served as a positive control. **g**, psNeuMigs, neutrophils and platelets were normalized with total protein and subjected to western blot analysis using antibodies against the indicated molecules. **h**, Diagram of the procedure for the digestion of purified migrasomes by proteinase K (PK) and subsequent incubation with s-plasma. **i**–**k**, CESs (**i**), platelets (**j**) or nsNeuMigs (**k**) were isolated from mice and digested with 100 μg ml^−1^ PK at 37 °C for 30 min, then incubated with s-plasma at 37 °C for 1 h. The samples (pre-digestion (ctrl), PK digested (PK), PK digested then s-plasma incubated (PK_s-pla) and s-plasma (s-pla)) were subjected to western blot analysis using antibodies against the indicated molecules. The grey values for the western blots were quantified using ImageJ. Source numerical data and unprocessed blots are available in Source Data Fig. 2. NoDiff, no difference. Source data

**Extended Data Fig. 2:**
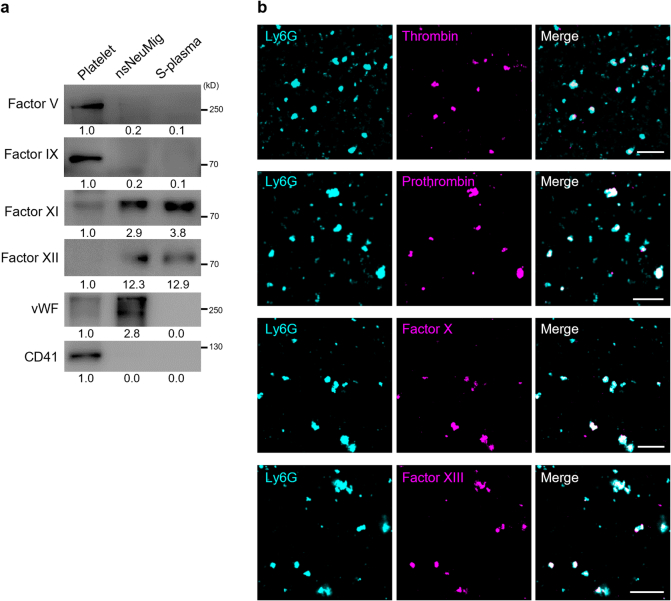
Coagulation factors are enriched in neutrophil-derived migrasomes. **a**, Western blot analysis of coagulation factors in platelets, nsNeuMig, and s-plasma. S-plasma is the supernatant after centrifuging plasma at 20,000 g for 1 hour. **b**, Dragonfly confocal microscopy images of nsNeuMigs revealed by immunofluorescence staining (IF). These results come from the same experiments as Fig. 2e. Migrasomes were negatively isolated and fixed with 4% paraformaldehyde for IF using anti-Ly6G and anti-thrombin, anti-prothrombin, anti-factor X and anti-factor XIII. Scale bar, 10 μm. Western blot gray values were quantified by Image J. Source unprocessed blots are available in source data. Source data

Next, we carried out quantitative mass spectrometry analysis on neutrophil-derived migrasomes and platelets isolated from platelet-depleted and neutrophil-depleted mice, respectively. Quantitative mass spectrometry analysis revealed that migrasomes are enriched with myeloperoxidase, a marker to neutrophils, in comparison to platelets. (Fig. 2a,b). Surprisingly, the mass spectrometry analysis revealed that neutrophil-derived migrasomes are highly enriched with coagulation factors, including prothrombin, factor XIII B, factor X, factor VIII, factor XI, factor XII and Von Willebrand factor, compared with platelets (Fig. 2a,b). To verify the enrichment of coagulation factors in neutrophil-derived migrasomes, we performed western blot analysis on the platelets from neutrophil-depleted mice and on both positively and negatively immune-purified migrasomes from platelet-depleted mice. To our surprise, the CD41^+^ platelets contained very low levels of coagulation factors (Fig. 2c,d). In contrast, Ly6G^+^ neutrophil-derived migrasomes purified by positive or negative selection were enriched with prothrombin, thrombin, factor XIII, factor VIII, factor X, factor XI and factor XII (Fig. 2c,d and Extended Data Fig. 2a). Confocal imaging analysis revealed that thrombin, prothrombin, factor X and factor XIII were indeed colocalized on Ly6G^+^ neutrophil-derived migrasomess (Fig. 2e and Extended Data Fig. 2b). To test whether the thrombin enriched on neutrophil-derived migrasomes was active, we carried out a thrombin activity assay. We found that plasma and platelets possessed very little thrombin activity; in contrast, purified neutrophil-derived migrasomes contained considerable thrombin activity (Fig. 2f). Paradoxically, none of these neutrophil-derived migrasome-enriched coagulation factors could be detected in neutrophil cell bodies (Fig. 2g). This observation, along with the fact that these coagulation factors are known to be secreted by the liver and present in plasma^22–24^, raises the interesting possibility that neutrophil-derived migrasomes adsorb and enrich coagulation factors from plasma on their surface. To test whether these migrasomes can re-adsorb coagulation factors, we treated them with proteinase K to remove existing factors, then incubated them with plasma. Post-treatment, the factors were completely removed from crude migrasomes but could be re-adsorbed upon exposure to plasma (Fig. 2h,i). In contrast, proteinase K-treated platelets did not re-adsorb coagulation factors (Fig. 2j). This re-adsorption ability was also confirmed in negatively purified neutrophil-derived migrasomes (Fig. 2k).

### Cholesterol ester is key to neutrophil-derived migrasome factor adsorption

**Fig. 3:**
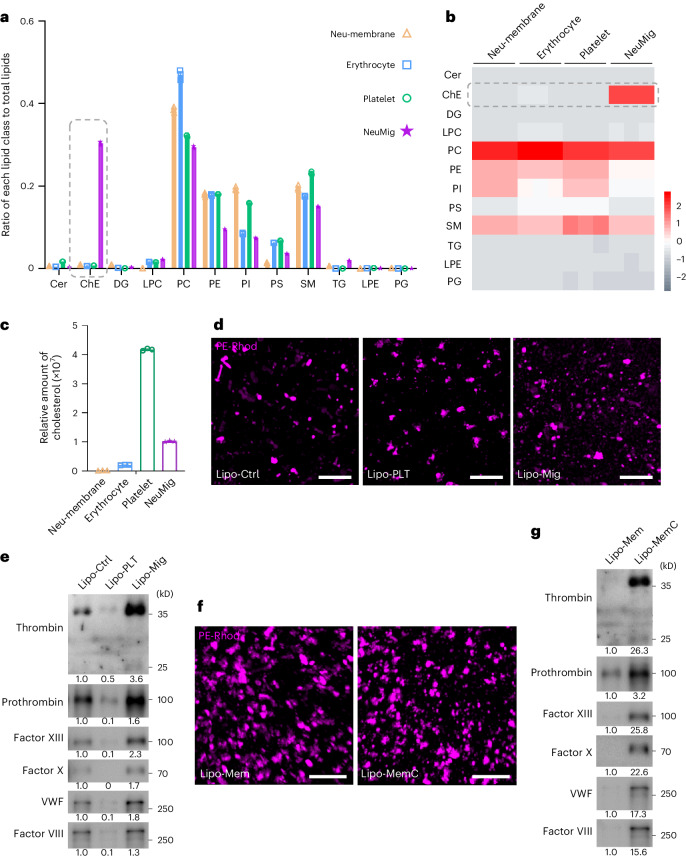
Cholesterol ester determines the ability of neutrophil-derived migrasomes to adsorb coagulation factors. **a**, Plot showing the differential abundance of lipids in plasma membranes of neutrophils (neu-membrane), erythrocytes, platelets and neutrophil-derived migrasomes. Total lipids were extracted from plasma membranes of neutrophils, erythrocytes, platelets and neutrophil-derived migrasomes and subjected to quantitative lipidomic analysis. The plot shows the ratio of each lipid to total lipids. *n* = 3 repeats. The data are presented as means ± s.e.m. **b**, Heat map of the distribution of lipids in plasma membranes of neutrophils, erythrocytes, platelets and neutrophil-derived migrasomes. **c**, Plot showing the differential abundance of cholesterol in plasma membranes of neutrophils, erythrocytes, platelets and neutrophil-derived migrasomes. *n* = 3 repeats. The data are presented as means ± s.e.m. **d**, Dragonfly confocal images of liposomes labelled with PE–Rhod (purple). Lipo-Mig contained PC, PE, PS, SM and ChE in similar ratios to neutrophil-derived migrasomes. Lipo-PLT was the same as Lipo-Mig except the ChE was replaced with cholesterol to mimic platelets. Lipo-Ctrl only contained PC, PE, PS and SM. Scale bars, 10 μm. **e**, Liposomes were incubated with s-plasma (37 °C for 1 h), then centrifuged down for western blot analysis using antibodies against coagulation factors. Numbers of liposomes (PE–Rhod^+^ vesicles) were counted by flow cytometry and the same number of each type of liposome was incubated with s-plasma. **f**, Dragonfly confocal images of liposomes labelled with PE–Rhod (purple). Lipo-Mem contained PC, PE, PS and SM in similar ratios to the plasma membranes of neutrophils. ChE was added to the composition of Lipo-Mem to make Lipo-MemC. Scale bars, 10 μm. **g**, Liposomes were incubated with s-plasma (37 °C for 1 h), then centrifuged down for western blot analysis using antibodies against coagulation factors. The numbers of liposomes (PE–Rhod^+^ vesicles) were counted by flow cytometry and the same number of liposomes from each sample was incubated with s-plasma. The western blot grey values were quantified using ImageJ. Source numerical data and unprocessed blots are available in Source Data Fig. 3. Cer, ceramide; DG, diglyceride; LPC, lysophosphatidylcholine; LPE, lysophosphatidylethanolamine; PC, phosphatidylcholine; PE, phosphatidylethanolamine; PG, phosphatidylglycerol; PI, phosphatidylinositol; PS, phosphatidylserine; SM, sphingomyelin; TG, triglyceride. Source data

The adsorption of coagulation factors by neutrophil-derived migrasomes suggests that their unique lipid composition could be a key factor. We isolated neutrophil-derived migrasomes from 300 platelet-depleted mice and conducted quantitative lipidomics after extracting their lipids. Control samples included plasma membranes from neutrophils, erythrocytes and platelets from neutrophil-depleted mice. The analysis showed similar levels of phosphatidylcholine, phosphatidylethanolamine, phosphatidylinositol, phosphatidylserine and sphingomyelin across all samples, but neutrophil-derived migrasomes were uniquely enriched with cholesterol ester (ChE), which was scarcely present in neutrophil plasma membranes or platelets. Conversely, platelets had higher cholesterol levels than neutrophil-derived migrasomes (Fig. 3a–c).

To determine whether the unique lipid composition—specifically ChE enrichment—influences coagulation factor adsorption, we prepared liposomes with varying lipid profiles. Lipo-Mig liposomes, mimicking neutrophil-derived migrasomes, contained phosphatidylcholine, phosphatidylethanolamine, phosphatidylserine, sphingomyelin and ChE in similar ratios. Lipo-PLT liposomes, mimicking platelets, were similar to Lipo-Mig liposomes but substituted ChE for cholesterol. As a control, Lipo-Ctrl contained only phosphatidylcholine, phosphatidylethanolamine, phosphatidylserine and sphingomyelin. After incubating these liposomes with mouse plasma and performing western blot analysis, we found that thrombin and other coagulation factors were notably enriched in Lipo-Mig compared with Lipo-Ctrl and Lipo-PLT (Fig. 3d,e). This suggests that ChE is crucial for the adsorption of coagulation factors by neutrophil-derived migrasomes. To simulate neutrophil plasma membranes, we created Lipo-Mem liposomes containing phosphatidylcholine, phosphatidylethanolamine, phosphatidylserine and sphingomyelin, matching neutrophil membrane ratios but without ChE. These liposomes failed to adsorb coagulation factors from plasma. However, introducing ChE to this formula, resulting in Lipo-MemC, enabled the liposomes to adsorb coagulation factors (Fig. 3f,g). This demonstrates that coagulation factors are not inherently packed within neutrophil-derived migrasomes but are adsorbed onto their surfaces, heavily influenced by the presence of ChE, which is abundant in these migrasomes but absent in neutrophil membranes and platelets.

### Neutrophil-derived migrasomes activate platelets in vitro

**Fig. 4:**
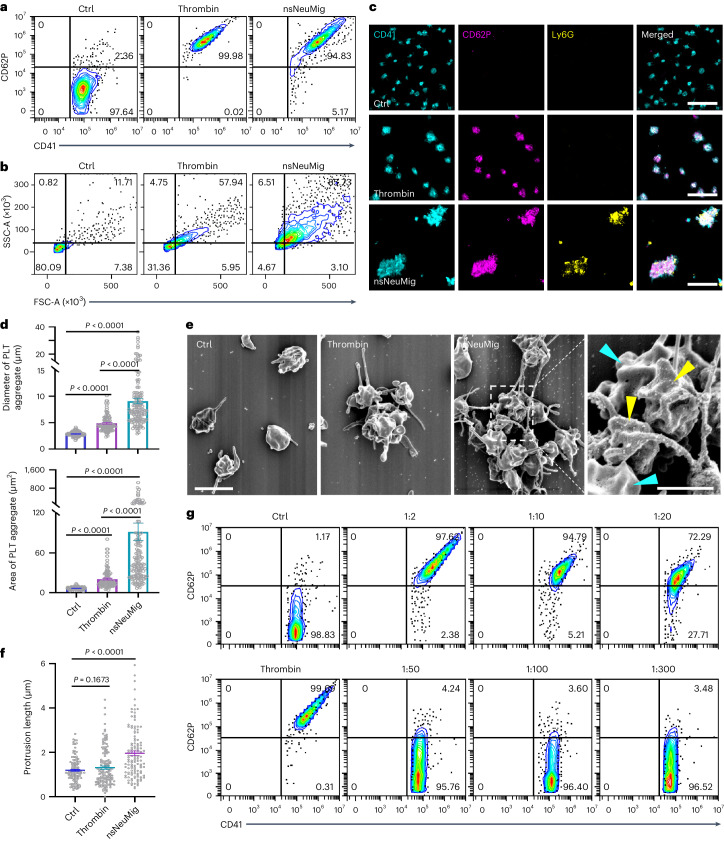
Neutrophil-derived migrasomes activate platelets in vitro*.* **a**,**b**, Flow cytometry analyses of platelet activation. Platelets were isolated from neutrophil-depleted mouse blood and stimulated with PBS, thrombin (1 U ml^−1^) or nsNeuMig (nsNeuMig:platelet = 1:2). Platelet activation is indicated by CD62P (**a**) and platelet morphology is indicated by SSC and FSC (**b**). **c**, Platelets activated by thrombin or neutrophil-derived migrasomes were stained with APC–anti-CD41 (cyan), PE–anti-CD62P (purple) and AF488–anti-Ly6G (yellow) and imaged by three-dimensional Dragonfly confocal microscopy. Scale bars, 20 μm. **d**, Measurement of the diameter and area of platelets (Ctrl) and platelet aggregates induced by thrombin and nsNeuMig. *n* = 104 platelets for Ctrl and *n* = 106 and *n* = 138 platelet aggregates for thrombin and nsNeuMig, respectively. **e**, SEM images of platelets activated in vitro by thrombin or nsNeuMig. Yellow arrowheads indicate migrasomes coated with anti-Ly6G-conjugated magnetic beads. Cyan arrowheads indicate platelets. Scale bar for the left three panels, 2 μm. The migrasomes and platelets in the dashed box are enlarged on the right (scale bar, 1 μm). **f**, Measurement of platelet protrusion length. *n* = 61 platelets for Ctrl, *n* = 64 platelets for thrombin and *n* = 62 platelets for nsNeuMig. **g**, Flow cytometry analysis of platelet activation. Platelets were isolated from neutrophil-depleted mouse blood and stimulated with PBS, thrombin or neutrophil-derived migrasomes (nsNeuMig:PLT = 1:2, 1:10, 1:20, 1:50, 1:100 or 1:300). Platelet activation is indicated by CD62P. All statistical data are presented as means ± s.e.m. *P* values were calculated using a two-tailed, unpaired *t*-test. Source numerical data are available in Source Data Fig. 4. Source data

**Extended Data Fig. 3:**
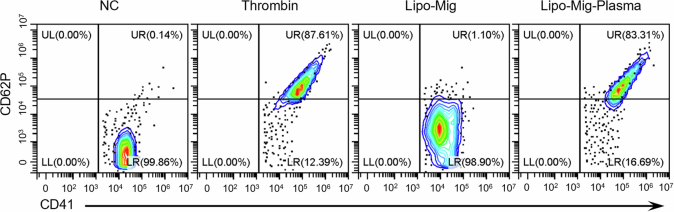
Migrasome-mimicking liposomes (Lipo-Mig) activate platelets *in vitro.* Flow cytometry analysis of platelet activation. Platelets were isolated from neutrophil-depleted mouse blood and stimulated with PBS, thrombin (1 U/mL), Lipo-Mig (1:2), or s-plasma-incubated Lipo-Mig. Platelet activation is indicated by CD62P. Source data

The abundance of neutrophil-derived migrasomes, their morphological resemblance to platelets and their coagulation factor enrichment led us to suggest that they may be involved in coagulation. To test this, we used an in vitro platelet activation assay where both thrombin and neutrophil-derived migrasomes effectively activated platelets, as shown by increased CD62P on the surface of platelets (Fig. 4a). Similarly, Lipo-Mig liposomes, mimicking neutrophil-derived migrasomes, activated platelets when incubated with plasma (Extended Data Fig. 3a). Notably, neutrophil-derived migrasomes induced substantial morphological changes in platelets, increasing both side scatter (SSC) and forward scatter (FSC), unlike thrombin, which caused only minor changes (Fig. 4b).

We visualized activated platelets via confocal microscopy, observing CD62P translocation to the surface, indicating activation by neutrophil-derived migrasomes. These migrasomes also formed larger aggregates with platelets than those induced by thrombin (Fig. 4c,d), corroborating the flow cytometry findings of enhanced scatter (Fig. 4b). SEM confirmed that neutrophil-derived migrasomes create larger platelet aggregates with more extensive filopodia (Fig. 4e,f). Using anti-Ly6G-conjugated magnetic beads showed that bead^+^ neutrophil-derived migrasomes form substantial aggregates with platelets (Fig. 4e). Effective platelet activation occurred at neutrophil-derived migrasome:platelet ratios of 1:2, 1:10 and 1:20, but not at 1:50 or lower (Fig. 4g).

### Neutrophil-derived migrasomes quickly accumulate at injury sites

**Fig. 5:**
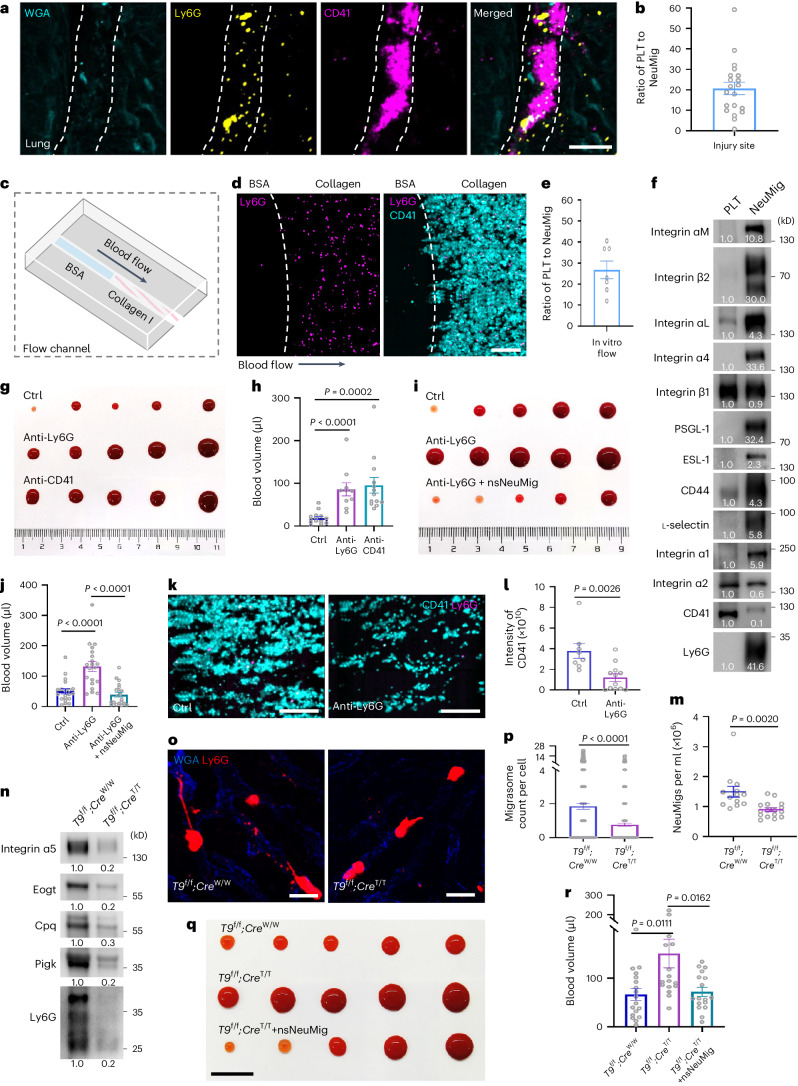
Neutrophil-derived migrasomes are essential for coagulation. **a**, Wounded lung imaging. AF488–WGA-labelled vessels (cyan), PE–anti-Ly6G (Gr1)-labelled neutrophil-derived migrasomes (yellow) and APC–anti-CD41-labelled platelets (purple) are shown. The dashed white lines indicate the wound boundary. Scale bar, 20 μm. **b**. Plot showing the ratio of platelets to neutrophil-derived migrasomes at injury sites. *n* = 20 fields of view. **c**, Diagram of the flow channel used for the in vitro flow assay. **d**, Dragonfly images of the flow assay. Scale bars, 50 μm. The dashed line indicates the boundary of collagen coating. **e**, Plot showing the ratio of platelets to neutrophil-derived migrasomes at collagen-coated sites. *n* = 7 mice. **f**, Western blot analysis of platelets and nsNeuMigs. **g**, Tail tip bleeding assay in control, neutrophil-depleted (anti-Ly6G) and platelet-depleted (anti-CD41) mice. Bloods were dripped on a clear plastic sheet for imaging. **h**, Statistical analysis of blood volume. *n* = 14 (Ctrl), 10 (anti-Ly6G) and 13 (anti-CD41) mice. **i**, Tail tip bleeding assay in control, neutrophil-depleted mice and mice injected with nsNeuMig (i.v.; 2 × 10^6^ per mouse). **j**, Statistical analysis of blood volume. *n* = 20 mice per group. **k**, Dragonfly images of in vitro flow assay using blood from control and neutrophil-depleted mice. Scale bars, 50 μm. **l**, Measurement of CD41 intensity in the flow channel. *n* = 8 (Ctrl) and 12 (anti-Ly6G) mice. **m**, Quantification of neutrophil-derived migrasomes in *Tspan9*^flox/flox^;*LysM-Cre*^WT/WT^ (*T9*^f/f^;*Cre*^*W/W*^) and *Tspan9*^flox/flox^;*LysM-Cre*^T/T^ (*T9*^f/f^;*Cre*^T/T^) mouse blood by imaging flow cytometry analysis. *n* = 13 (*T9*^f/f^;*Cre*^W/W^) and 16 (*T9*^f/f^;*Cre*^T/T^) mice. **n**, Western blot analysis of nsNeuMigs isolated from *T9*^f/f^;Cre^W/W^ and *T9*^f/f^;*Cre*^T/T^ mice. Samples from ten mice were pooled and analysed together. **o**, Intravital imaging of neutrophil-derived migrasomes in the livers of *T9*^f/f^;*Cre*^W/W^ and *T9*^f/f^;*Cre*^T/T^ mice. Scale bars, 20 μm. **p**, Quantification of neutrophil-derived migrasomes in *T9*^f/f^;*Cre*^W/W^ and *T9*^f/f^;*Cre*^T/T^ mice. *n* = 395 (*T9*^f/f^;*Cre*^W/W^) and 410 (*T9*^f/f^;*Cre*^T/T^) cells from six mice each. **q**, Tail tip bleeding assay in each group. Scale bar, 1 cm. **r**, Statistical analysis of blood volumes. *n* = 18 (*T9*^f/f^;*Cre*^W/W^) and 17 (other groups) mice. All statistical data are presented as means ± s.e.m, *P* values were calculated using a two-tailed, unpaired *t*-test. The data in **b**, **e**, **h**, **j**, **l**, **p** and **r** were pooled from three independent experiments. The grey values of the western blots were quantified using ImageJ. Source numerical data and unprocessed blots are available in Source Data Fig. 5. Source data

**Extended Data Fig. 4:**
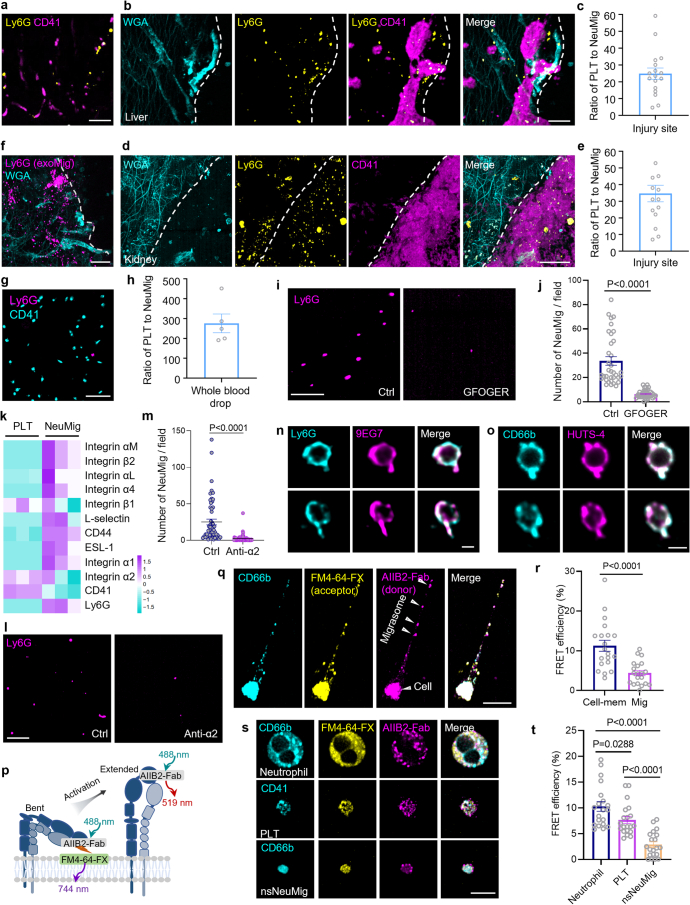
Neutrophil-derived migrasomes accumulate at injury site by directly adhere to the ECMs. **a, b, d**, Intravital imaging of non-wounded liver (**a**), wounded liver (**b**) or kidney (**d**). AF488-WGA labels blood vessels, PE-anti-Ly6G (Gr1) labels neutrophil-derived migrasomes, and APC-anti-CD41 labels platelets. Scale bar, 20 μm (**a**, **b**), 50 μm (**d**). The dashed-white-line indicates the wound boundary. **c, e**. Quantification of the ratio of platelets to neutrophil-derived migrasomes at the liver (**c**) or kidney (**e**) injury site. n = 17 mice (**c**), n = 15 field-of-views from 3 mice (**e**). **f**, Imaging of exogenous-injected neutrophil-migrasomes in wounded liver. Scale bar, 20 μm. **g**, Imaging of platelets and neutrophil-derived migrasomes within whole blood which were placed in non-coated channels. Scale bar, 20 μm. **h**, Quantification of the ratio of platelets to migrasomes in non-coated channels with no flow. n = 5 mice. **i, l**. Dragonfly images present the *in vitro* flow assay using ctrl and GFOGER (200 μg/mL for 30 min) (**i**) or anti-integrin α2 (HMα2) (5 μg/mL for 25 min) (**l**) treated blood. Scale bar, 10 μm. **j, m**. Statistical analysis of the number of neutrophil-derived migrasomes accumulated at collagen exposing site in each group. n = 35 (Ctrl) 40 (GFOGER) 55 (Ctrl) 57 (anti-integrin α2) field-of-views. **k**, Heat map of the distribution of adhesion molecules in platelets and neutrophil-derived migrasomes revealed by quantitative mass spectrometry analysis. **n-o**. Confocal images of nsNeuMigs revealed by immunofluorescence staining. Scale bar, 1 μm. **p**. Diagram showing how the FRET assay assess the conformation of integrins. **q, s**. Confocal images of neutrophils and *in vitro* generated migrasomes (**q**), or isolated human neutrophil-derived migrasomes, neutrophils and platelets (**s**) revealed by immunofluorescence staining using anti-integrin β1 (AIIB2-Fab), anti-CD66b and FM4-64-FX before bleach. Scale bar, 10 μm (**q**), 5 μm (**s**). **r, t**. FRET measurement of the samples in **q** and **s**. n = 20 for each group. All of the statistical data are presented as the mean ± s.e.m, *P* values were calculated using the two-tailed, unpaired t-test. Data were pooled from six (**c**), three (**e**, **j**, **k**), and four (**m**) independent experiments. Source numerical data are available in source data. Source data

To investigate the role of neutrophil-derived migrasomes in coagulation in vivo, we first checked whether circulating migrasomes could be deposited at the site of injury in a manner similar to platelets. To do this, we first cut a shallow wound on mouse liver, lung or kidney, then we carried out imaging of the wound and the surrounding non-wounded area. To label the neutrophil-derived migrasomes, we injected fluorophore-conjugated anti-Ly6G into blood vessels. Similarly, we labelled platelets using fluorophore-conjugated anti-CD41. We found that in the control area, the platelets and neutrophil-derived migrasomes were floating and rapidly moving with the bloodstream (Extended Data Fig. 4a). In contrast, several minutes after wounding, neutrophil-derived migrasomes were concentrated on the wound in large numbers and platelets had already aggregated (Fig. 5a and Extended Data Fig. 4b,d). We also introduced exogenous neutrophil-derived migrasomes by intravenous (i.v.) injection. The exogenous neutrophil-derived migrasomes were also rapidly enriched at the injury site (Extended Data Fig. 4f). Put together, these data suggest that neutrophil-derived migrasomess in the circulation can quickly accumulate at injury sites with kinetics similar to platelets.

### Neutrophil-derived migrasomes at injury sites trigger coagulation

As discussed above, the neutrophil-derived migrasome:platelet ratio was 1:300—far below the activation threshold. We hypothesized that this ratio reaches the activation threshold at injury sites. To quantify this ratio at the injury site, we calculated the number of neutrophil-derived migrasomes by counting the number of Ly6G^+^ vesicles at the injury site. Due to aggregation of platelets at the wound, it is difficult to quantify the precise number of platelets in a similar way. Therefore, we measured the mean CD41 intensity of single platelets and the total CD41 intensity of aggregated platelets and roughly calculated the number of platelets at the injury site. We found that the average neutrophil-derived migrasome:platelet ratios at injury sites were 1:20.7 (lung), 1:24.9 (liver) and 1:34.7 (kidney) (Fig. 5a,b and Extended Data Fig. 4b–e). These results indicate that the neutrophil-derived migrasome/platelet ratio indeed reaches the activation threshold at injury sites (Fig. 4g), implying that neutrophil-derived migrasomes are preferentially recruited to wounds.

To further test this hypothesis, we set up an in vitro thrombus formation flow assay using a flow channel under arterial flow conditions (1,000 s^−1^). To mimic injury, the flow channel was partially coated with collagen^25,26^ (Fig. 5c). Mouse blood was collected and stained with a conjugate of phycoerythrin and Ly6G antibody (PE–anti-Ly6G) and a conjugate of allophycocyanin and CD41 antibody (APC–anti-CD41) and perfused over the flow channel. We found that platelets and neutrophil-derived migrasomes only accumulated in the collagen-coated region, and the average neutrophil-derived migrasome:platelet ratio in the aggregates was 1:26.8 (Fig. 5d,e). Again, the ratio reached the threshold of platelet activation (Figs. 4g and Fig. 5a,b and Extended Data Fig. 4b–e). In contrast, the neutrophil-derived migrasome:platelet ratio was 1:276 in a non-coated channel with no flow, reflecting this ratio in physiological blood (Extended Data Fig. 4g,h). Additionally, we found that neutrophil-derived migrasomes no longer adhered to the collagen-coated site after pre-incubation with the collagen-mimetic peptide (GFOGER)^27,28^ (Extended Data Fig. 4i,j), confirming that neutrophil-derived migrasomes accumulate at injury sites by directly binding to collagen. It is worth noting that although both neutrophil-derived migrasomes and platelets are enriched on collagen, these migrasomes have a greater degree of enrichment than platelets.

Next, we asked why neutrophil-derived migrasomes preferentially accumulate on injury sites or on collagen-coated regions. It is well established that platelet deposition is mediated by integrins and other adhesion molecules, which bind to collagen and other adhesion receptors that are exposed at injury sites. Our previous publication found that integrins are highly enriched on migrasomes^7^. Mass spectrometry data revealed that, compared with platelets, neutrophil-derived migrasomes are enriched with most of the adhesion molecules, including integrin α_1_, Mac-1 (integrin α_M_/β_2_), lymphocyte function-associated antigen-1 (LFA-1) (integrin α_L_/β_2_), very late antigen-4 (VLA-4) (integrin α_4_/β_1_), l-selectin, CD44 and E-selectin ligand 1 (Extended Data Fig. 4k). Western blot analysis confirmed the presence or enrichment of adhesion molecules such as integrins (α_M_, β_2_, α_L_, α_4_, β_1_, α_1_ and α_2_), l-selectin, CD44, E-selectin ligand 1 and P-selectin glycoprotein ligand-1 (PSGL-1) on neutrophil-derived migrasomes (Fig. 5f). Using the integrin α_2_ blocking antibody, we further confirmed that interaction between collagen and integrin α_2_ is essential for the accumulation of neutrophil-derived migrasomes (Extended Data Fig. 4l,m). Although not tested, it is likely that other adhesion molecules also contribute to the accumulation of neutrophil-derived migrasomes at injury sites. These data may explain why neutrophil-derived migrasomes show preferential enrichment at wounds compared with platelets.

The mere presence of integrins on a cell surface does not conclusively demonstrate that these molecules mediate adhesive interactions with a substrate. The conformational changes of integrins to a high-affinity state must be tightly regulated. To evaluate the state of integrins on neutrophil-derived migrasomes, we employed antibodies that specifically recognize activated integrin β_1_ (refs. ^7,29^). Confocal imaging revealed that the localized integrin β_1_ on neutrophil-derived migrasomes was in its activated, ligand-binding state (Extended Data Fig. 4n,o). To delve deeper into the activation state of integrins on neutrophil-derived migrasomes, we utilized fluorescence resonance energy transfer (FRET) assay to examine the orientation of the integrin ectodomain relative to the membrane^30,31^ (Extended Data Fig. 4p). The results were consistent, with the FRET efficiency of neutrophil-derived migrasomes being significantly lower than that of platelets and neutrophils in vitro and in vivo (Extended Data Fig. 4q–t). This indicates that the ectodomain of integrin β_1_ on neutrophil-derived migrasomes is more extended than that on platelets and neutrophils. These results suggest that the integrins on neutrophil-derived migrasomes are in a high-affinity active state and capable of direct interaction and binding to substrates. In summary, our findings demonstrate that neutrophil-derived migrasomes are enriched with high-affinity-state adhesion molecules. Consequently, they would rapidly and preferentially adhere and accumulate at injury sites, thereby initiating the coagulation cascade.

### Exogenous neutrophil-derived migrasomes reduce bleeding in neutrophil loss

**Extended Data Fig. 5:**
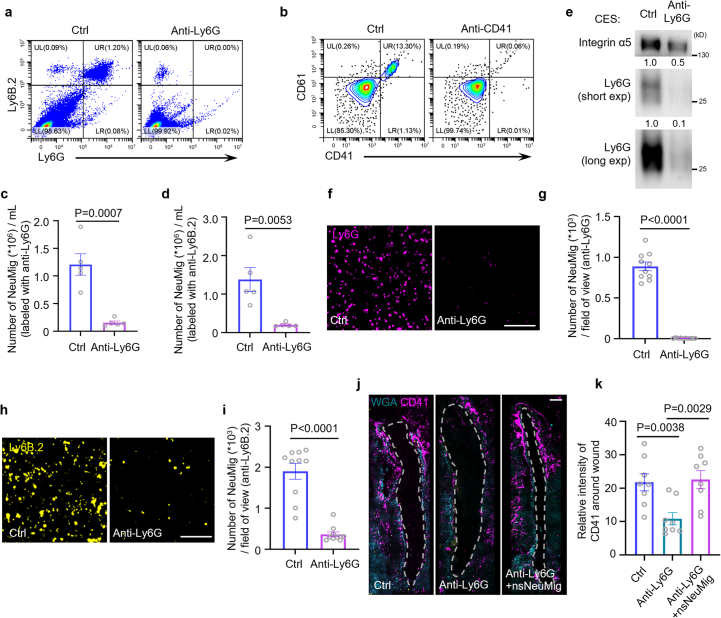
Neutrophil-depletion lead to the dramatic decrease of neutrophil-derived migrasomes. **a-b**, Flow cytometry analysis of whole blood cells from control mice and neutrophil-depleted mice (**a**) or platelet-depleted mice (**b**) after staining with FITC-anti-Ly6B.2 and PE-anti-Ly6G (**a**), or FITC-anti-CD61 and APC-anti-CD41 (**b**). Samples from five mice were pooled and analyzed together. **c-d**, Quantification of the number of neutrophil-derived migrasomes in ctrl and neutrophil-depleted mice revealed by imaging-flow cytometry analysis. Mouse blood samples were collected and stained with AF488-anti-Ly6G, PE-anti-CD41 and AF647-anti-Ly6B.2 for analysis. n = 5 mice for each group. **e**, Crude migrasomes were isolated from the same volume of blood from control or neutrophil-depleted mice, and then analyzed by western blotting using antibodies against integrin α5 and Ly6G. **f**, Dragonfly confocal images of crude migrasomes stained with PE-anti-Ly6G. Scale bar, 20 μm. **g**, Quantification of the number of neutrophil-derived migrasomes in images of crude migrasomes stained with PE-anti-Ly6G. Samples were pooled from 5 mice for each group. n = 10 field-of-views for each group. **h**, Dragonfly confocal images of crude migrasomes stained with Alexa Fluor 647-anti-Ly6B.2. Scale bar, 20 μm. **i**, Quantification of the number of neutrophil-derived migrasomes in images of crude migrasomes stained with AF647-anti-Ly6B.2. Samples were pooled from 5 mice for each group. n = 10 field-of-views for each group. **j**, Stitch Dragonfly confocal imaging of liver wounds in control mice, neutrophil-depleted (anti-Ly6G) mice, and neutrophil-depleted mice rescued with nsNeuMigs (i.v. 2 × 10^6^ per mouse). AF488-WGA labels vessels; APC-anti-CD41 labels platelets. Scale bar, 200 μm. Dashed white lines indicate the wound boundaries. **k**, Statistical analysis of the relative fluorescence intensity of CD41 (platelets) enriched around the wound boundaries; n = 8 mice for each group. All of the statistical data are presented as the mean ± s.e.m, *P* values were calculated using the two-tailed, unpaired t-test. Gray values of western blots were quantified by Image J. Source numerical data and unprocessed blots are available in source data. Source data

Next, we tested the role of neutrophil-derived migrasomes in coagulation in vivo. First, we depleted the neutrophils in mice with anti-Ly6G antibody. FACS analysis confirmed that the vast majority of neutrophils were depleted, whereas the number of platelets was not affected (Extended Data Figs. 1e and 5a). As a control, we also depleted the platelets with anti-CD41 antibody and confirmed the platelet depletion by FACS (Extended Data Fig. 5b). Similarly, depletion of platelets did not affect the number of neutrophils (Extended Data Fig. 1e). We verified that the number of neutrophil-derived migrasomes was indeed greatly decreased in neutrophil-depleted mice by multiple means, including imaging flow cytometry, western blot and confocal imaging (Extended Data Fig. 5c–i). We then assessed the effect of neutrophil depletion on coagulation using the tail tip bleeding assay, with bleeding volume as the measurement. We found that depletion of neutrophils or platelets significantly increased the bleeding volume, and the bleeding volume in neutrophil-depleted mice was similar to that in platelet-depleted mice (Fig. 5g,h). These observations suggest that neutrophils play an essential role in coagulation. Next, we tested the role of neutrophil-derived migrasomes in coagulation by injecting purified migrasomes from wild-type mice into neutrophil-depleted mice. We found that exogenous neutrophil-derived migrasomes can rescue impaired coagulation in neutrophil-depleted mice and exogenous neutrophil-derived migrasomes can significantly reduce the bleeding volume in neutrophil-depleted mice to below the bleeding volume in control mice (Fig. 5i,j). When neutrophil-depleted blood was subjected to in vitro thrombus formation assay, only a few neutrophil-derived migrasomes were left and the number of aggregated platelets was markedly decreased (Fig. 5k,l). Together, these lines of evidence further support the essential role of neutrophil-derived migrasomes in coagulation.

Next, we checked clot formation on the wound in control and neutrophil-depleted mice. In neutrophil-depleted mice, the platelets cannot form a platelet plug on the wound even though the number of platelets is normal (Extended Data Figs. 1e and 5j,k). Adding back exogenous neutrophil-derived migrasomes largely restored the platelet plug formation in neutrophil-depleted mice (Extended Data Fig. 5j,k). This suggests that neutrophil-derived migrasomes are required for platelet plug formation in vivo.

### *Tspan9* regulates coagulation via neutrophil-derived migrasome formation

**Extended Data Fig. 6:**
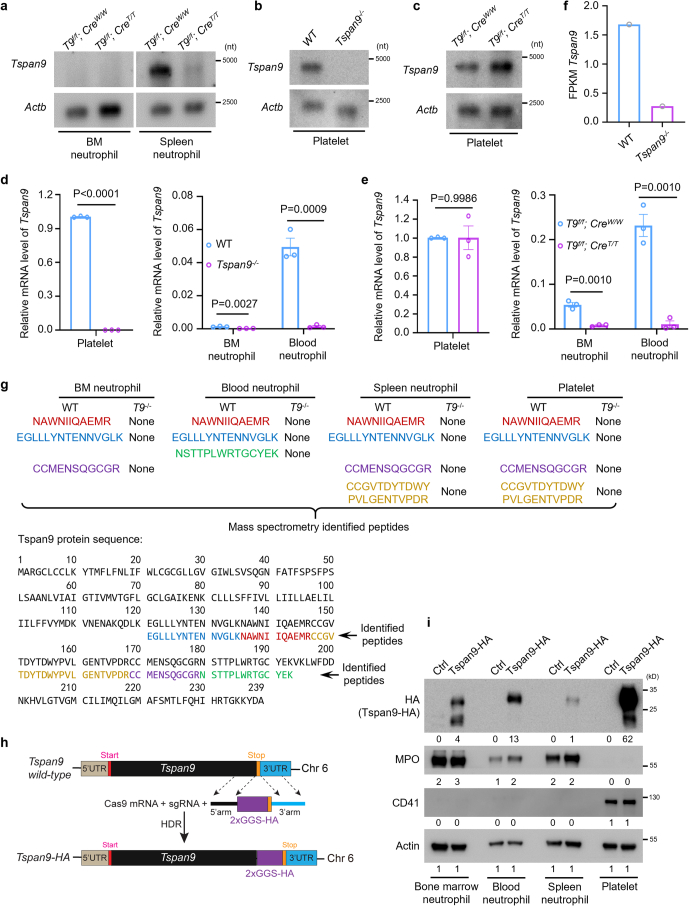
*Tspan9* is expressed in neutrophils. **a-c**, Northern blot analysis of *Tspan9* and *Actb* in mRNAs extracted from platelets, bone marrow neutrophils and spleen neutrophils isolated from WT, *Tspan9*^-/-^, *T9*^f/f^; *Cre*^W/W^ and *T9*^f/f^; *Cre*^T/T^ mice. Northern blotting was performed on mRNA extracted from 3.6 μg platelet total RNA and 10 μg neutrophil total RNA. **d**, Q-PCR analysis of *Tspan9* in platelets and neutrophils isolated from WT and *Tspan9*^-/-^ mice. n = 3 independent experiments. **e**, Q-PCR analysis of *Tspan9* in platelets and neutrophils isolated from *T9*^f/f^; *Cre*^W/W^ and *T9*^f/f^; *Cre*^T/T^ mice. n = 3 independent experiments. **f**, RNA sequencing analysis of *Tspan9* in mRNAs extracted from WT blood neutrophils and *Tspan9*^-/-^ blood neutrophils. Samples from 5 mice were pooled and analyzed together. **g**. Mass spectrometry analysis of neutrophils and platelets. Bone marrow neutrophils, blood neutrophils, spleen neutrophils and platelets were isolated from WT and *Tspan9*^-/-^ mice respectively and subjected to mass spectrometry analysis. The identified peptides were validated specifically aligned with *Tspan9* using BLAST searching in UniProt and NCBI database. **h**. Diagram of the HA tag knock-in strategy at the C-terminus of *Tspan9*. **i**. Western blot analysis of platelets and various neutrophils isolated from control and *Tspan9*-HA knock-in mice using indicated antibodies. Gray values of western blots were quantified by Image J. The gray values are normalized to actin. All statistical data are presented as the mean ± s.e.m. *P* values indicated in plots were calculated using the two-tailed, unpaired t-test. Source numerical data and unprocessed blots are available in source data. Source data

Previously, we reported that members of the tetraspanin family regulate migrasome formation^6,8^ and neutrophils from *Tspan9*^−/−^ mice have impaired migrasome formation^9^. Northern blotting (Extended Data Fig. 6a–c), quantitative PCR (Extended Data Fig. 6d,e), RNA sequencing (Extended Data Fig. 6f), mass spectrometry analysis (Extended Data Fig. 6g) and *Tspan9-HA* knock-in mice (Extended Data Fig. 6h,i) revealed that *Tspan9* is expressed in neutrophils isolated from the blood and spleen (Extended Data Fig. 6a–i).

**Extended Data Fig. 7:**
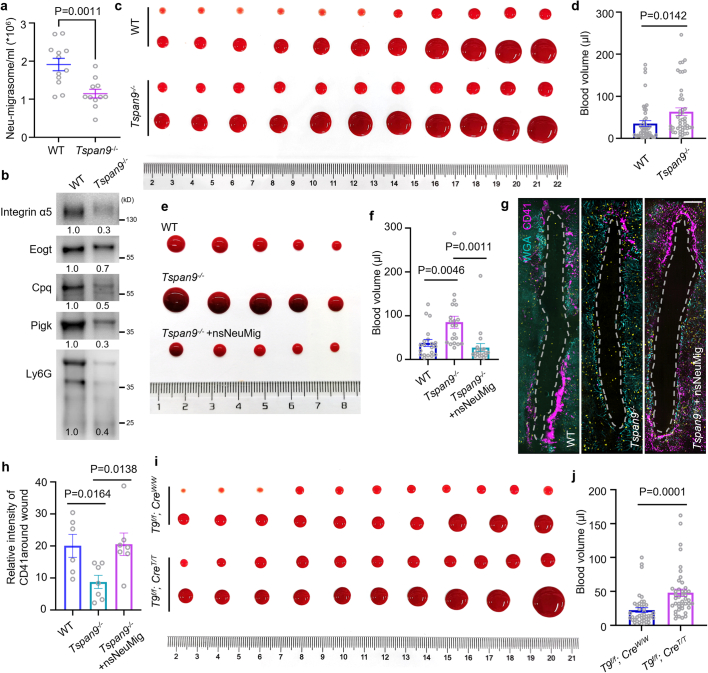
*Tspan9* regulates neutrophil-derived migrasomes and is involved in coagulation. **a**, Quantification of neutrophil-derived migrasomes in blood from WT and *Tspan9*^-/-^ mice by imaging-flow cytometry analysis; n = 12 mice for WT, n = 11 mice for *Tspan9*^-/-^. **b**, Western blot analysis of nsNeuMigs isolated from the same volume blood of WT and *Tspan9*^-/-^ mice using antibodies against the indicated molecules. Samples from 10 mice were pooled and analyzed together. Gray values of western blots were quantified by Image J. **c**, Tail-tip bleeding assay in WT and *Tspan9*^-/-^ mice. Blood was dripped onto a clear plastic sheet for imaging. **d**, Statistical analysis of blood volumes. Data were pooled from four independent experiments. n = 45 mice for each group. **e**, Tail-tip bleeding assay in WT, *Tspan9*^*-/-*^ and nsNeuMig-injected (i.v. 2 × 10^6^ per mouse) mice. Blood was dripped onto a clear plastic sheet for imaging. **f**, Statistical analysis of bleeding volume. Data were pooled from four independent experiments. n = 20 mice for each group. **g**, Stitch imaging of liver wounds in WT, *Tspan9*^*-/-*^ and nsNeuMig-injected (i.v. 2 × 10^6^ per mouse) mice. AF488-WGA labels vessels; PE-anti-Ly6G (Gr1) labels neutrophils and migrasomes; APC-anti-CD41 labels platelets. Scale bar, 200 μm. Dashed white lines indicate the wound boundaries. **h**, Statistical analysis of relative fluorescence intensity of CD41 (platelets) enriched around the wound boundaries. n = 6 mice for the WT group; n = 7 mice for the *Tspan9*^-/-^ and *Tspan9*^-/-^ + nsNeuMig groups. **i**, Tail-tip bleeding assay in *T9*^f/f^; Cre^W/W^ and *T9*^f/f^; Cre^T/T^ mice. Blood was dripped on a clear plastic sheet for imaging. **j**, Statistical analysis of bleeding volumes. Data were pooled from four independent experiments. n = 43 mice in *T9*^f/f^; Cre^W/W^ group; n = 42 mice in *T9*^f/f^; Cre^T/T^ group. All statistical data are presented as the mean ± s.e.m. *P* values were calculated using the two-tailed, unpaired t-test. Source numerical data and unprocessed blots are available in source data. Source data

Using imaging flow cytometry analysis, we confirmed that the number of neutrophil-derived migrasomes is indeed reduced in *Tspan9*^−/−^ mice (Extended Data Fig. 7a). To verify the reduction of these migrasomes in the blood of *Tspan9*^−/−^ mice by an alternative method, we purified migrasomes from equal volumes of blood from wild-type or *Tspan9*^−/−^ mice. We then carried out western blot analysis using antibodies against Ly6G (the marker for neutrophils) and various migrasome-enriched proteins, including integrin α_5_, Cpq, Pigk and Eogt (Aer61)^12^. In the migrasome fraction isolated from *Tspan9*^−/−^ mouse blood, the levels of these migrasome-enriched proteins were reduced (Extended Data Fig. 7b), consistent with a lower number of neutrophil-derived migrasomes in *Tspan9*^−/−^ mice. Furthermore, the bleeding volumes were significantly increased in *Tspan9*^−/−^ mice, suggesting that coagulation was impaired in these mice (Extended Data Fig. 7c–f). Adding exogenous neutrophil-derived migrasomes can rescue coagulation impairment (Extended Data Fig. 7e,f). Imaging analysis showed that platelet plug formation at the wound site was impaired in *Tspan9*^−/−^ mice and adding neutrophil-derived migrasomes could restore platelet plug formation in the wound (Extended Data Fig. 7g,h). To further confirm these results and rule out any effects caused by *Tspan9* knockout in other cell types, we generated *Tspan9* conditional knockout mice using the Cre-LoxP system. *Tspan9*^flox/flox^ mice were generated and crossed with *LysM-Cre* mice to obtain mice with myeloid cell lineage-specific knockout of *Tspan9*. Using imaging flow cytometry and western blot analysis, we confirmed that the number of neutrophil-derived migrasomes was indeed reduced in *Tspan9*^flox/flox^;*LysM-Cre*^T/T^ mice (Fig. 5m,n). Intravital imaging of *Tspan9*^flox/flox^;*LysM-Cre*^WT/WT^ and *Tspan9*^flox/flox^;*LysM-Cre*^T/T^ mice confirmed that *Tspan9*^flox/flox^;*LysM-Cre*^T/T^ mice have impaired neutrophil-derived migrasome formation (Fig. 5o,p). Again, we found that the bleeding volumes were significantly increased in *Tspan9*^flox/flox^;*LysM-Cre*^T/T^ mice (Fig. 5q,r and Extended Data Fig. 7i,j), suggesting that coagulation was impaired in these mice. Moreover, adding exogenous neutrophil-derived migrasomes could rescue the coagulation impairment (Fig. 5q,r), further supporting the essential role of these migrasomes in coagulation.

### Human neutrophil-derived migrasomes contribute to activation of coagulation

**Fig. 6:**
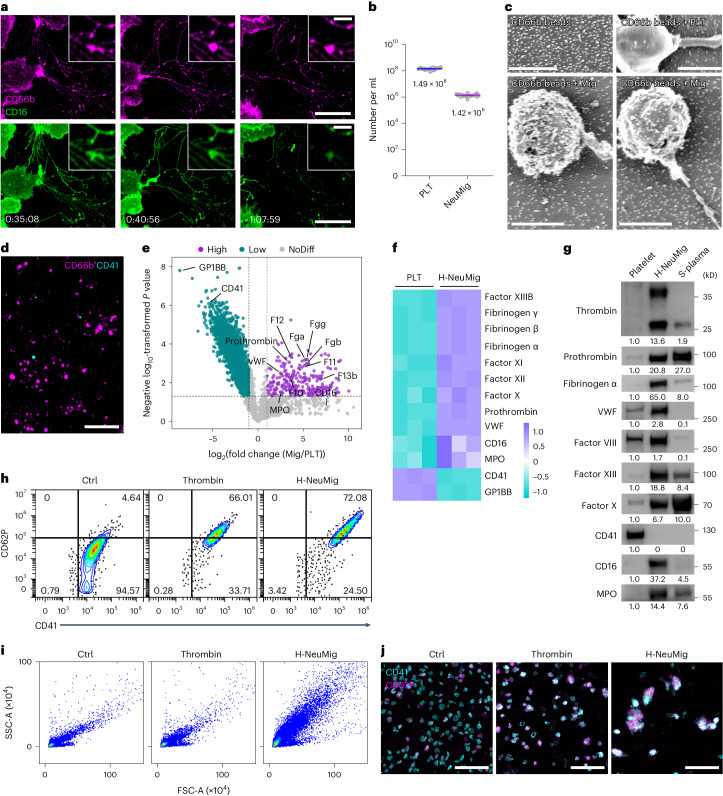
Human neutrophil-derived migrasomes play essential roles in coagulation. **a**, Time-lapse imaging of neutrophils isolated from human blood. Neutrophils were seeded in dishes coated with fibronectin (10 μg ml^−1^ for 30 min) and labelled with APC–anti-CD16 and PE–anti-CD66b. Time interval, 45 s. Scale bars, 20 μm and 3 μm (inserts). **b**, Quantification of neutrophil-derived migrasomes and platelets in human peripheral blood. Human whole blood samples were stained with PE–anti-CD66b and APC–anti-CD41 for imaging flow cytometry analysis. The gating strategy was the same as for Fig. 1d. *n* = 20 humans. The data are presented as means ± s.e.m. **c**, SEM images of neutrophil-derived migrasomes isolated from human peripheral blood by positive selection using anti-CD66b-conjugated microbeads (Miltenyi Biotec). Anti-CD66b microbeads (top left) and platelets incubated with anti-CD66b microbeads (top right) served as controls. Scale bars, 1 μm. **d**, Neutrophil-derived migrasomes were isolated from human peripheral blood by negative selection, then stained with PE–anti-CD66b (purple) and APC–anti-CD41 (cyan) for imaging using the Dragonfly microscopy system. Scale bar, 10 μm. **e**, Volcano plot showing the differential abundance of proteins in isolated human neutrophil-derived migrasomes versus human platelets. The migrasomes and platelets were isolated and subjected to label-free quantitative mass spectrometry analysis. The purple dots represent a migrasome/platelet abundance ratio of ≥2 (*P* < 0.05) and the cyan dots represent a migrasome/platelet abundance ratio of ≤0.5 (*P* < 0.05). *n* = 3 biologically independent experiments. *P* values were calculated using a two-tailed, unpaired *t*-test. **f**, Heat map of the distribution of coagulation factors in human platelets and human neutrophil-derived migrasomes. **g**, Western blot analysis of coagulation factors and marker proteins for platelets or neutrophil-derived migrasomes in human platelets, human nsNeuMig (H-NeuMig) and human s-plasma. **h**,**i**, Flow cytometry analyses of platelet activation. Platelets were isolated from human peripheral blood and stimulated with PBS, thrombin (1 U ml^−1^) or H-NeuMig (Mig:PLT = 1:2). Platelet activation is indicated by CD62P (**h**) and platelet morphology is indicated by SSC and FSC (**i**). **j**, Platelets activated by thrombin or H-NeuMig were stained with the indicated antibodies and imaged by Dragonfly microscopy. Scale bars, 20 μm. The grey values of the western blots were quantified using ImageJ. Source numerical data and unprocessed blots are available in Source Data Fig. 6. Source data

To test whether human neutrophil-derived migrasomes also contribute to the activation of coagulation, we carried out experiments using human blood neutrophils and neutrophil-derived migrasomes isolated from human blood. In mice, we used Ly6G to label the migrasomes; unfortunately, there is no homologue of Ly6G in humans. To find a suitable marker with which to label human migrasomes, we screened a panel of surface markers for human neutrophils. We found that both CD66b and CD16 antibodies can label migrasomes on human neutrophils. The CD16 antibody signal fades away quickly after migrasome formation, while the CD66b antibody gives a strong and long-lasting signal on migrasomes (Fig. 6a and Supplementary Video 2). Using the anti-CD66b antibody, we performed detailed live image analysis of migrasome formation in in vitro-cultured human primary neutrophils. Similar to mouse neutrophils, neutrophils from human blood migrated very fast and produced a lot of CD66b^+^ migrasomes (Fig. 6a and Supplementary Video 2).

Next, we calculated the ratio of neutrophil-derived migrasomes to platelets in humans. For this purpose, we collected adult peripheral blood from healthy volunteers and performed imaging flow analysis using PE–anti-human CD66b and APC–anti-human CD41, which labels human platelets. The number of CD66b^+^ neutrophil-derived migrasomes was about 1/100 of the number of platelets (Fig. 6b).

To test whether human neutrophil-derived migrasomes are enriched with coagulation factors, we isolated these migrasomes and platelets from the peripheral blood of healthy human donors (Fig. 6c,d). We then carried out quantitative mass spectrometry analysis on isolated human neutrophil-derived migrasomes and platelets. We found that, similar to the results in mice, human neutrophil-derived migrasomes are highly enriched with coagulation factors, including prothrombin, factor XIII B, factor X, factor XI, factor XII, Von Willebrand factor and fibrinogens, compared with platelets (Fig. 6e,f). Western blot analysis on the platelets and migrasomes isolated from the human blood showed that the CD41^+^ platelets contained very low levels of coagulation factors, whereas the migrasomes were enriched with thrombin, prothrombin, factor XIII, factor VIII and factor X (Fig. 6g).

Next, we carried out an in vitro human platelet activation assay. We found that both thrombin and human neutrophil-derived migrasomes strongly activated platelets (Fig. 6h). Interestingly, the migrasomes greatly increased the SSC and FSC of platelets, while thrombin caused only very minor changes to the SSC and FSC (Fig. 6i). Confocal imaging revealed that the platelet aggregates induced by human neutrophil-derived migrasomes are notably larger than the aggregates induced by thrombin (Fig. 6j), consistent with the enhanced FSC and SSC detected by flow cytometry (Fig. 6i). These data are consistent with our findings in mice and support the essential role of neutrophil-derived migrasomes in coagulation.

### Infection or inflammation boosts neutrophil-derived migrasome formation

**Fig. 7:**
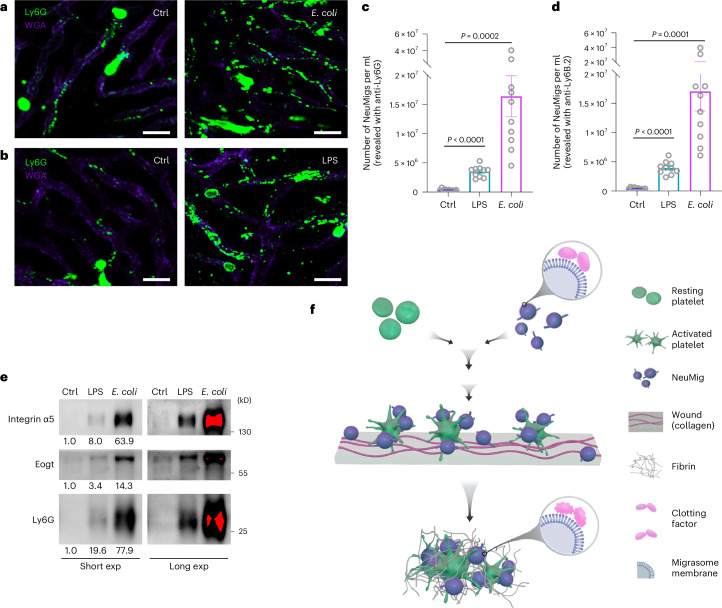
Neutrophil-derived migrasome formation is markedly enhanced under infection or inflammation conditions. **a**,**b**, Intravital imaging of neutrophils and neutrophil-derived migrasomes in the livers of control mice or mice infected with *E. coli* (i.p. injection of 1 × 10^9^ c.f.u. in **a**) or treated with LPS (i.p. injection of 10 mg kg^−1^ LPS in **b**) for 3–4 h. Neutrophils and neutrophil-derived migrasomes were labelled with PE–anti-mouse Ly6G (Gr1; green) and blood vessels were labelled with AF647–WGA (purple). Scale bars, 20 μm. **c**,**d**, Quantification of blood neutrophil-derived migrasomes in control, LPS-treated (i.p.; 10 mg kg^−1^) and *E. coli-*infected (i.p.; 1 × 10^9^ c.f.u.) mice using Amnis imaging flow cytometry analysis. Blood was collected 4 h after LPS or *E. coli* injection from the orbital sinus and stained with PE–anti-CD41 and either AF488–anti-Ly6G (**c**) or AF647–anti-Ly6B.2 (**d**) for analysis. *n* = 10 mice per group. The data are presented as means ± s.e.m. *P* values were calculated using a two-tailed, unpaired *t*-test. **e**, Neutrophil-derived migrasomes were negatively isolated from the same volume of blood from control, LPS-treated (i.p.; 10 mg kg^−1^) or *E. coli*-infected (i.p;. 1 × 10^9^ c.f.u.) mice and analysed by western blotting using antibodies against marker proteins for migrasomes. Ten mice per group were studied. Samples were pooled per group for subsequent analysis. The grey values of the western blots were quantified using ImageJ. **f**, Diagram showing how neutrophil-derived migrasomes are involved in the coagulation system. Source numerical data and unprocessed blots are available in Source Data Fig. 7. exp, exposure. Source data

It is well known that neutrophil release from the bone marrow is substantially elevated during infection or inflammatory insult^32,33^. To test whether the number of neutrophil-derived migrasomes changes after infection, we infected mice with *Escherichia coli* by intraperitoneal (i.p.) injection. Since it is well established that bacterial infection can induce an inflammatory response, we also treated mice with lipopolysaccharide (LPS), which is the major component of Gram-negative bacterial cell walls and can cause an acute inflammatory response. Using intravital imaging, we found that the number of neutrophil-derived migrasomes was dramatically increased after *E. coli* infection or LPS treatment (Fig. 7a,b). Imaging flow analysis and western blot analysis confirmed that the number of Ly6G^+^ neutrophil-derived migrasomes was significantly increased in blood from *E. coli*-infected mice and LPS-treated mice (Fig. 7c–e). It is worth noting that *E. coli* infection caused a significantly greater enhancement of the neutrophil-derived migrasome number than LPS. Thus, factors other than LPS in *E. coli* may also contribute to activation of neutrophil-derived migrasome formation. Together, these data suggest that infection or inflammation can dramatically enhance neutrophil-derived migrasome formation. These data also prompt us to speculate that neutrophil-derived migrasome formation is part of the immune response to infection.

## Discussion

In this study, we discovered that neutrophil-derived migrasomes circulating in the bloodstream are rich in adhesion molecules and coagulation factors, aiding clot formation at injury sites. Reducing these migrasomes, by depleting neutrophils or knocking out *Tspan9*, leads to excessive bleeding that is reversible with exogenous neutrophil-derived migrasomes. These migrasomes adsorb coagulation factors in a ChE-dependent manner that is similar in both human and mouse blood and effectively activate platelets. Increased neutrophil-derived migrasome production, triggered by bacterial infection and systemic inflammation, suggests their role in immune responses. Overall, our findings highlight neutrophil-derived migrasomes as crucial to the coagulation system (Fig. 7f). Moreover, our data indicate that their numbers increase significantly with bacterial infections and systemic inflammation. These findings suggest that they could be involved in various thrombotic disorders, potentially serving as a therapeutic target and early diagnostic marker for these conditions.

Neutrophil-derived migrasomes, similar in size and morphology to platelets and enriched with integrins, probably share platelets’ flow dynamics but have superior adhesive properties, enhancing their attachment to injury sites. By adsorbing active thrombin, they act as coagulation catalysts. They form a binary system with platelets, segregating catalysts and substrates into separate compartments (Fig. 7f). This system prevents accidental coagulation activation under normal conditions, yet ensures rapid response to injury.

Previous research has reported normal coagulation in *Tspan9*^−/−^ mice^34^. However, our findings indicate significant coagulation impairment in both *Tspan9*^−/−^ and *Tspan9*^flox/flox^;*LysM-Cre*^T/T^ mice, as confirmed by extensive testing across both strains. Future studies should explore this discrepancy. In addition, cholesterol esters, which are typically sequestered in intracellular lipid droplets due to their hydrophobic nature, are unusually enriched in neutrophil-derived migrasomes, enhancing their ability to bind coagulation factors. Although these esters are generally scarce in the plasma membrane, they can be incorporated into giant unilamellar vesicles, altering membrane properties significantly^35^. The mechanism of their accumulation in neutrophil-derived migrasomes remains unclear, suggesting an unknown process unique to their membrane’s biophysical characteristics. Further research is needed to elucidate this phenomenon.

## Methods

This study complied with all of the relevant ethical regulations. All animal experiments were approved by the Institutional Animal Care and Use Committee and conducted in accordance with governmental and Tsinghua University guidelines for animal welfare (permission numbers 18-YL2 and 22-YL2). The use of human blood samples was approved by the ethics committee on human research at the West China Hospital of Sichuan University (permission number 2023 Review (no. 4 and no. 1546)) and Institutional Review Board of Tsinghua University (project number 20220193). Informed consent was obtained from the participants. No compensation was offered to any participant.

### Mice

C57BL/6J mice were used in this study. When relevant and applicable, age- and sex-matched mice were randomly chosen from the same cages to be included in the experimental and control groups. All C57BL/6J mice used in this study were obtained from the animal centre of Tsinghua University. Mice were housed in ventilated cages in a specific pathogen-free animal facility under a 12 h light/12 h dark cycle at 20–26 °C and 40–70% humidity.

*Tspan9*^−/−^ mice were generated by CRISPR–Cas9 in the C57BL/6J background. The single guide RNA sequence used for CRISPR–Cas9 was 5′-GAAGGTGGCGAAGTTGCCTT-3′. Mice were genotyped by PCR with the following primers: *Tspan9*-KO forward (GCTGCCTCGTCCCATTTACT) and *Tspan9*-KO reverse (ACGCTGAGAAGCAGACACTT).

*Tspan9*^flox/flox^ mice were generated with technical expertise from GemPharmatech. Age- and sex-matched mice between 6 and 12 weeks of age were used. Cell-specific deletion of the *Tspan9* allele was obtained by Cre-mediated recombination after crossing with *LysM-Cre* mice. Mice were genotyped by PCR with the following primers: 5′ arm *Tspan9* forward (TGTCGGTACTCAATACATATTGGCTGA), 5′ arm *Tspan9* reverse (ATCCATAGAACGAGTGGGCCTGTAAA), 3′ arm *Tspan9* forward (AAACAGCATGGCACCCAGAGACA) and 3′ arm *Tspan9* reverse (CACAGCTTGACCCACAAAGCCAT).

*LysM-Cre* mice were kind gifts from X. Hu’s laboratory at Tsinghua University. The primers used were as follows: *LysM-Cre* primer 1 (CCCAGAAATGCCAGATTACG), *LysM-Cre* primer 2 (CTTGGGCTGCCAGAATTTCTC) and *LysM-Cre* primer 3 (TTACAGTCGGCCAGGCTGAC).

The *Tspan9-HA* mouse model was generated through cytoplasmic microinjection of two-cell-stage embryos by J.L.’s laboratory at the Shanghai Institute of Biochemistry and Cell Biology. To efficiently generate the *Tspan9* knock-in mouse model, we adopted the two-cell homologous recombination CRISPR method published in recent work^36^. The *Tspan9-HA* single guide RNA sequence was 5′-AAGTACGACGCCTGAGGCTG-3′.

### Human blood

Human bloods were collected from healthy volunteers of 18–60 year of age with no regard for gender. There was no self-selection bias. Blood samples were processed in the research laboratories.

### Intravital imaging

Male C57BL6/J mice of 8–12 weeks of age were used. For neutrophil imaging, conjugates of Alexa Fluor 647 dye (AF647) and Wheat Germ Agglutinin (WGA; W32466; Thermo Fisher Scientific) (that is, AF647–WGA; 1–3 μg) and phycoerythrin and Ly6G (anti-Gr1) antibody (12-5931-82; eBioscience) (that is, PE–anti-Ly6G; 0.5–1.0 μg) were injected intravenously into the mice. Then, anaesthesia was induced in the mice by i.p. injection of avertin (375 mg kg^−1^). Subsequently, the mice were dissected to expose the liver on a plate with a cover glass in the centre for spinning disk imaging.

For wound imaging, mice received i.v. injections of AF488–WGA (5 μg; W11261; Thermo Fisher Scientific), APC–anti-CD41 (0.5–1.0 μg; 133914; BioLegend) and PE–anti-Ly6G (Gr1) (0.5–1.0 μg). After anaesthesia was induced, the mice were dissected to expose the organ, then a small shallow wound was cut in the organ with scissors. The mice were placed on a plate with a cover glass in the centre for Dragonfly spinning disk imaging.

### Imaging flow cytometry analysis

Blood was diluted four times with 20 mM ethylenediaminetetraacetic acid (EDTA) containing phosphate-buffered saline (PBS) and stained with PE–anti-Ly6G and APC–anti-CD41 (mouse) or PE–anti-CD66b and APC–anti-CD41 (human). After 15–20 min, a fourfold volume of PBS was added for imaging streaming analysis. An ImageStream Mark II flow cytometer (Luminex) was used to image the streamed cells, platelets or migrasomes and Inspire software was used for data acquisition. Some 300,000 Ly6G^+^ or CD41^+^ events (mouse) and 500,000 total events (human) were obtained. IDEAS software (Luminex) was used for data analysis according to the gating strategy.

### Isolation of CESs from mouse blood

For platelet-depleted mice, mice were i.p. injected with 0.8 mg kg^−1^ anti-CD41 antibody (553847; BD Pharmingen) prepared in 200 µl PBS. After 12–18 h, blood was collected from the ocular venous plexus in collection buffer (PBS supplemented with 40 mM EDTA) and the blood mixture (blood:buffer = 1:1) was then centrifuged at 800*g* and 4 °C for 10 min to remove the blood cells and finally at 20,000*g* and 4 °C for 40–60 min. The pellet was the CES fraction.

### Positive selection of neutrophil-derived migrasomes from mouse blood

The CESs purified from platelet-depleted mice were resuspended with PBS (supplemented with 2% s-plasma (that is, the supernatant after centrifuging plasma at 20,000*g* and 4 °C for 1 h)) and incubated with anti-Ly-6G MicroBeads (130-120-337; Miltenyi Biotec) for 60 min at 4 °C. The bead-treated migrasomes were then subjected to positive selection using a DynaMag-Spin (12320D; Invitrogen) for more than 12 h at 4 °C. The supernatant was removed and washed very gently three times with PBS (supplemented with 2% s-plasma). The PBS was then removed to obtain the positively sorted neutrophil-derived migrasome preparation.

### Negative selection of neutrophil-derived migrasomes from mouse blood

A total of 25–30 ml mouse blood was used per batch of experiments. The CESs purified from platelet-depleted mice (normal or infected) were resuspended with 60 μl PBS (supplemented with 5% rat serum) and subjected to negative selection using an EasySep Mouse Neutrophil Enrichment Kit (19762; STEMCELL Technologies). In brief, 20 μl Biotin anti-mouse Ter119 (116204; BioLegend) and 20 μl Enrichment Cocktail were added and the mixture was incubated at 4 °C for 15 min. The sample was centrifuged at 20,000*g* and 4 °C for 30 min and the supernatant was removed. The pellet was resuspended with 60 μl PBS (5% rat serum was added) and incubated with 40 μl Biotin Selection Cocktail at 4 °C for 15 min. Magnetic particles (80 μl) were then added and incubated at 4 °C for 60 min with gentle mixing three to four times during incubation. Then, 400 μl PBS was added and the tube was placed into the DynaMag-Spin (12320D; Invitrogen) and incubated for 10–20 min at 4 °C. The suspension was transferred to a new blocked tube and centrifuged at 20,000*g* and 4 °C for 30–60 min to obtain the negatively sorted neutrophil-derived migrasome preparation.

### Negative selection of neutrophil-derived migrasomes from human blood

Human blood was obtained from hospital and the blood mixture was then centrifuged at 800*g* and 4 °C for 5 min, followed by 1,000*g* and 4 °C for 15 min to remove the blood cells. The supernatant was collected and diluted three times with PBS (supplemented with 40 mM EDTA) and subjected to centrifugation at 20,000*g* and 4 °C for 60–90 min. The pellet was the CES fraction. A total of 20 ml human plasma (60 ml after dilution) was used per batch of experiments. The CESs were resuspended with 60 μl PBS (supplemented with 5% rat serum) and subjected to negative selection using an EasySep Human Neutrophil Isolation Kit (17957; STEMCELL Technologies) combined with an EasySep Mouse Neutrophil Enrichment Kit (19762; STEMCELL Technologies). In brief, 20 μl Biotin anti-human CD235ab (306618; BioLegend) and 20 μl Biotin anti-human CD41 (303734; BioLegend) were added and the mixture was incubated at 4 °C for 15 min. The sample was centrifuged at 20,000*g* and 4 °C for 30 min and the supernatant was removed. The pellet was resuspended with 60 μl PBS (5% rat serum was added) and incubated with 30 μl Biotin Selection Cocktail (from kit 19762) at 4 °C for 10 min and then further incubated with 30 μl Human Neutrophil Isolation Cocktail (from kit 17957) at 4 °C for 15 min. Then, 40 μl Magnetic Particles (from kit 19762) were added and incubated at 4 °C for 10 min, then further incubated with 40 μl Dextran RapidSpheres (from kit 17957) at 4 °C for 60 min with gentle mixing three to four times during incubation. Next, 400 μl PBS was added and the tube was placed into the DynaMag-Spin (12320D; Invitrogen) and incubated for 10–20 min at 4 °C. The suspension was transferred to a new blocked tube and centrifuged at 20,000*g* and 4 °C for 30–60 min to obtain the human negatively sorted neutrophil-derived migrasome preparation.

### Purification of platelets from blood

Mouse blood (1 ml) was collected into a tube containing 4% sodium citrate (pH 7.2; 125 μl) and mixed gently. Platelets were purified from mouse blood according to a previously published protocol^37^. For human platelet isolation, 10 μM Prostaglandin E1 (HY-B0131; MedchemExpress) was added to the blood as soon as possible to prevent platelet activation. And a similar method with the purification of mouse platelet was used.

### Scanning electron microscopy

Purified migrasomes or platelets were placed on a silicon wafer that was coated with poly-l-lysine (P4707; Sigma–Aldrich; 1 h at room temperature) for more than 4 h, then fixed with 2.5% glutaraldehyde and 2% paraformaldehyde for more than 1 h. After washing with 0.1 M PB buffer (100 ml: 36 ml 0.2 M Na_2_HPO_4_•12H_2_O, 14 ml 0.2 M NaH_2_PO_4_•2H_2_O and 50 ml distilled water (pH 7.2)), samples were washed in PB buffer for 10 min, then incubated with 1% osmium tetroxide/1.5% potassium ferricyanide for 30 min. After washing in distilled water, samples were dehydrated in an ethanol series (50, 70, 80, 90, 100, 100 and 100% for 2 min each). The samples were dried in a critical point dryer (Leica EM CPD300; Leica Microsystems). A 10 nm gold layer was sputtered onto the surface of the samples, which were then observed under an FEI Helios NanoLab G3 UC scanning electron microscope.

### Flow cytometry sorting and analysis

Mouse blood was collected from the ocular venous plexus and put into a tube containing blood collection buffer (blood:buffer = 1:1). The blood mixture was centrifuged at 800*g* and 4 °C for 5 min and the cell pellet was resuspended with ammonium–chloride–potassium lysis buffer for 2 min to lyse the red blood cells. The lysate was then centrifuged at 1,000*g* and 4 °C for 5 min and the supernatant was removed. The pellet was resuspended with PBS and stained with PE–anti-Ly6G and APC–anti-CD41 at room temperature for 15 min and then centrifuged at 1,000*g* and 4 °C for 5 min to obtain the blood cell mixture. The cell mixture was resuspended with PBS for flow cytometry sorting by MoFlo Astrios EQ or MoFlo XDP (Beckman Coulter) and imaging by Dragonfly spinning disk microscopy (Andor).

For blood migrasome analysis, blood migrasomes were purified and stained with AF647–anti-Ly6G at room temperature for 15 min. AF647 Rat IgG2a served as a staining control. The migrasome mixture was centrifuged at 20,000*g* and 4 °C for 30 min. The migrasome pellet was resuspended with PBS for flow cytometry analysis using a CytoFLEX LX (Beckman Coulter).

### Quantitative proteomics analysis

A total of 30 μg protein per sample was used for proteomics analysis. After reduction with 10 mM tris(2-carboxyethyl)phosphine and alkylation with 40 mM chloroacetamide, protein samples were digested using trypsin and lysC (protein:enzyme = 100:1) at 37 °C overnight. Peptides were desalted using C18 StageTips and dried by SpeedVac, then resuspended in 0.1% formic acid in H_2_O for mass spectrometry. The liquid chromatography–tandem mass spectrometry instrument used here was an UltiMate 3000 RSLCnano system directly interfaced with an Orbitrap Fusion Lumos Tribrid mass spectrometer from Thermo Fisher Scientific. Peptides were loaded into a trap column (75 µm × 20 mm; 3 µm; C18; 100 Å; 164535; Thermo Fisher Scientific) with a maximum pressure of 620 bar using mobile phase A (0.1% formic acid in H_2_O), then separated on an analytical column (100 μm inner diameter; packed in house with ReproSil-Pur C18-AQ 1.9 μm resin from Dr. Maisch) with a gradient of 6–55% using mobile phase B (80% acetonitrile and 0.08% formic acid) at a flow rate of 250 nl min^−1^ for 120 min. A field asymmetric ion mobility spectrometry (FAIMS) device was also used for peptide separation. It was placed between the nano-electrospray source and the mass spectrometer. The FAIMS separation settings were as follows: mode = standard resolution; carrier gas flow = 4 l min^−1^; and total carrier gas flow = static. The compensation voltages of FAIMS were −45 and −60 V. The mass spectrometry data were acquired in data-independent acquisition mode and there was a single full-scan mass spectrum in the orbitrap (350–1,650 *m/z*; resolution = 120,000 at 200 *m/z*) with an automatic gain control target value of 2 × 10^6^, followed by multiple tandem mass spectrometry spectra in a cycle time of 3 s. Fragmentation was performed via a normalized collision energy of 35% with automatic gain control of 5 × 10^5^ and a maximum injection time of 100 ms. Precursor peptides were isolated with 33 variable windows spanning from 300 to 1,500 *m/z* at a resolution of 30,000. The data-independent acquisition mass spectrometry data were analysed using the Spectronaut 15.6 software applying default settings, in which quantitation was based on the MS2 area and data filtering was set to *Q*-value sparse. The database used was UniProt Mouse (downloaded on 4 January 2021; 17,056 sequences).

### Migrasome and platelet digestion assay

Blood migrasomes and platelets were isolated from mouse blood and each sample was divided into three equal parts. The first part served as a control. The second and third parts were digested with proteinase K (0706; Amresco; 100 μg ml^−1^) at 37 °C for 30 min, then washed with five times the volume of PBS and centrifuged at 2,000*g* and 4 °C for 5 min (platelets) or 20,000*g* and 4 °C for 40 min (migrasomes) to obtain the digested pellet. The third part was resuspended and incubated with 500 μl s-plasma at 37 °C for 60 min. The mixture was centrifuged and the pellet was washed once with PBS and centrifuged to obtain the samples. The three parts of platelets and migrasomes and s-plasma were lysed with 8 M urea for western blot analysis.

### Platelet activation in vitro

Purified platelets were resuspended with PBS supplemented with 5% foetal bovine serum to 1 million μl^−1^. Some 10 million purified platelets in up to 80 µl reaction buffer (422201; BioLegend) were placed into three tubes. PBS, purified migrasomes (or liposomes) and thrombin (T4648; Sigma–Aldrich; 1 U ml^−1^) were added respectively and mixed with the platelets for 30 min at room temperature. After 30 min, PE–anti-CD62P, APC–anti-CD41 and AF488–anti-Ly6G were added and incubated with the samples for 15 min at room temperature in the dark. Then, 100 μl PBS was added to the tubes and the samples were divided into three parts. The first part was used for flow cytometry analysis. The second and third parts were fixed with the mix of 2.5% glutaraldehyde and 2% paraformaldehyde for Dragonfly imaging and SEM imaging.

### Depletion of platelets and neutrophils

To deplete platelets, mice were intraperitoneally injected with 0.6–1.0 mg kg^−1^ anti-CD41 antibody (553847; BD Biosciences) prepared in 200 μl PBS 12–18 h before the experiments. To deplete neutrophils, mice were injected intraperitoneally with an initial 200 μg followed by 100 μg three times weekly of InVivoPlus anti-Ly6G antibody (BP0075-1; BioXCell). InVivoPlus rat IgG2a (BP0089; BioXCell) served as a control.

### Tail tip bleeding assay

Mice were anaesthetized by i.p. injection of avertin (375 mg kg^−1^) and placed on a flat plate. The terminal 6 mm was cut off the tail. The clipped tail was immediately immersed in warm PBS (100 μl) supplemented with 40 mM EDTA and allowed to bleed for 20 min. The blood was mixed well with the PBS and the bleeding volume was measured using the collected total volume minus the volume of PBS. The remaining blood mixture was dropped onto a clear plastic film and photographed.

### Quantitative lipidomics analysis

The ultra-performance liquid chromatography system was coupled to a Q Exactive HF-X orbitrap mass spectrometer (Thermo Fisher Scientific) equipped with a heated electrospray ionization probe. The lipid extracts were separated by Cortecs C18 100 mm × 2.1 mm Waters column. In the binary solvent system, mobile phase A consisted of acetonitrile:H_2_O (60:40) and 10 mM ammonium acetate whereas mobile phase B consisted of isopropanol:acetonitrile (90:10) and 10 mM ammonium acetate. The flow rate was 250 μl min^−1^ and the gradient time was 35 min. The column chamber and sample tray were held at 40 and 10 °C, respectively. Data with mass ranges of 240–2,000 and 200-2,000 *m/z* were collected using data-dependent tandem mass spectrometry in positive and negative ion modes, respectively. Full-scan and fragment spectra were collected at resolutions of 60,000 and 15,000, respectively. The source parameters were as follows: spray voltage = 3,000 V; capillary temperature = 320 °C; heater temperature = 300 °C; sheath gas flow rate = 35 a.u.; and auxiliary gas flow rate = 10 a.u. Data analysis and lipid identification were performed using the software LipidSearch (Thermo Fisher Scientific). All molecular identifications were based on tandem mass spectrometry, with an MS1 mass error of <5 ppm and an MS2 mass error of <8 ppm.

### Liposome preparation

Lipids, including 1-palmitoyl-2-oleoyl-glycero-3-phosphocholine (phosphatidylcholine), 1,2-dioleoyl-*sn*-glycero-3-phosphoethanolamine (phosphatidylethanolamine), 1-palmitoyl-2-oleoyl-*sn*-glycero-3-phospho-l-serine (sodium salt) (phosphatidylserine), cholesteryl oleate (ChE), sphingomyelin (Brain, Porcine) (sphingomyelin), cholesterol and 1,2-dioleoyl-*sn*-glycero-3-phosphoethanolamine-*N*-(lissamine rhodamine B sulfonyl) (Rhod–PE), were purchased from Avanti Polar Lipids. Lipids were mixed according to the following recipes, where the percentage represents the mass ratio: for Lipo-Mig, 34.60% ChE, 33.51% phosphatidylcholine, 10.82% phosphatidylethanolamine, 4.03% phosphatidylserine and 17.05% sphingomyelin; for Lipo-PLT, 34.60% cholesterol, 33.51% phosphatidylcholine, 10.82% phosphatidylethanolamine, 4.03% phosphatidylserine and 17.05% sphingomyelin; and for Lipo-Ctrl, as for Lipo-Mig except that ChE was removed. Each liposome sample was supplemented with 0.1% Rhod–PE for imaging or flow cytometry analysis. The lipid mixtures were blow dried with a nitrogen stream and further air dried for 30 min at 37 °C. The lipid film was then hydrated completely with HEPES buffer (10 mM HEPES (pH 7.4) and 140 mM NaCl) to obtain multilamellar liposomes. To obtain unilamellar liposomes, multilamellar liposomes were subjected to 15 cycles of freezing in liquid nitrogen and thawing in a 42 °C water bath, then the liposomes were extruded 20 times through a 1,000 nm pore size polycarbonate film to produce small unilamellar vesicles. The number of liposomes (Rhod–PE^+^ vesicles), including multilamellar liposomes and unilamellar liposomes, was counted by flow cytometry.

### Liposome–plasma incubation

For plasma preparation, bloods were collected in sodium heparin-coated tubes and subjected to centrifugation at 2,000*g* and 4 °C for 5 min. Supernatants were collected and subjected to centrifugation at 100,000*g* and 4 °C for 30 min. The supernatants were collected (S100 plasma) for incubation with liposomes.

For liposome preparation, liposomes were counted using flow cytometry and normalized with the number of Rhod–PE^+^ vesicles.

Each liposome sample (~1 mg; normalized with the same number) was incubated with 600 μl S100 plasma at 37 °C for 1 h. Then, the mixtures were diluted fivefold with HEPES buffer (10 mM HEPES (pH 7.4) and 140 mM NaCl) and subjected to centrifugation at 100,000*g* and 4 °C for 60 min. The pellets were collected as pellet 1 and the supernatants were collected and subjected to centrifugation at 20,000*g* and 4 °C for 60 min. The pellets were collected and combined with pellet 1 to obtain the total pellets. The total pellets were washed twice with HEPES buffer. Pellets were lysed with 8 M urea for western blot analysis.

### Northern blotting

Neutrophil or platelet total messenger RNAs were run on 1% formaldehyde agarose gels and then transferred to Hybond-N+ nylon membrane (RPN303B; Amersham). Subsequently, membranes were crosslinked with ultraviolet light at 120 mJ cm^−2^ and then pre-hybridized at 68 °C with hybridization buffer (AM8669; Ambion) for 1 h. For *Tspan9*, pre-hybridized membranes were hybridized overnight at 70 °C with biotin-labelled probe. *Actin* probe was hybridized at 65 °C. Blots were then washed and hybridized membranes were exposed to horseradish peroxidase-conjugated streptavidin (89880; Thermo Fisher Scientific). The membranes were visualized with a chemiluminescence detection system (Bio-Rad) after incubation with horseradish peroxidase substrates (34094; Thermo Fisher Scientific).

The probe for detecting *Tspan9* was transcribed in vitro (AM1354; Thermo Fisher Scientific) using Biotin-16-UTP (11388908910; Roche). The template for in vitro transcription was complementary DNA containing the T7 promotor, amplified with the following primers: forward primer (5′-TAATACGACTCACTATAGGGAGAGTGACCATTCGGGATGC-3′) and reverse primer (5′-CAGGTAAAAAGTACGACGCC-3′). The mixed probes for detecting *Actin* were directly transcribed in vitro (10881767001; Roche) using Biotin RNA Labeling Mix (11685597910; Roche) with the following sequences: 5′-TAATACGACTCACTATAGGGTCCTGCTCGAAGTCTAGAGCAACATAGCACAGCTTCTCTTTGATGTCACG-3′ and 5′-TAATACGACTCACTATAGGGTTGGCATAGAGGTCTTTACGGATGTCAACGTCACACTTCATGATGGAATT-3′.

### In vitro flow assay

A flow channel was partially (the right half) coated with 200 μg ml^−1^ collagen (C8062; Solarbio) overnight at room temperature. The whole channel was then blocked with 5% bovine serum albumin (BSA) (A8010; Solarbio), which was diluted in HEPES buffer for 1 h at room temperature. The channel was washed with 1 U ml^−1^ heparin (9041-08-1; Sigma–Aldrich) containing Hanks’ Balanced Salt Solution buffer (with Ca^2+^ and Mg^2+^). Mouse bloods were collected in heparin-coated tubes. Some 300 μl whole blood was incubated with PE–anti-Ly6G (1A8 and Gr1) and APC–anti-CD41 for 5 min and placed in a 2 ml syringe for perfusion over the flow channel at a shear rate of 1,000 s^−1^ using a syringe pump. Then, the flow channel was perfused with HEPES buffer supplemented with 1 U ml^−1^ heparin for 5 min at 1,000 s^−1^ before Dragonfly imaging.

### Quantitative PCR with reverse transcription

To detect *Actb*, the forward primer was 5′-GGCTGTATTCCCCTCCATCG-3′ and the reverse primer was 5′-CCAGTTGGTAACAATGCCATGT-3′. To detect *Tspan9*, the forward primer was 5′-CAAGGCAACTTCGCCACCTTCT-3′ and the reverse primer was 5′-GGAAGCCCGTCACCATGACGAT-3′.

### Immunofluorescence staining of neutrophil-derived migrasomes

Neutrophil-derived migrasomes were fixed with 4% paraformaldehyde in 1.5 ml tubes for 15 min and washed three times with PBS. For each wash, the migrasomes were centrifuged at 20,000*g* and 4 °C for 5–20 min to remove the supernatant. Neutrophil-derived migrasomes were blocked with 1% BSA for 30 min then incubated at room temperature for 2 h with primary antibodies against the indicated coagulation factors (primary antibodies were diluted 1:200 in PBS supplemented with 1% BSA and 0.05% saponin). Samples were washed twice with PBS and then incubated with secondary antibodies (diluted 1:500 in PBS supplemented with 1% BSA) at room temperature for 2 h. Samples were washed twice with PBS and resuspended with PBS. The stained samples were dropped in a glass-bottom confocal dish and placed at 4 °C overnight, then observed with Dragonfly confocal microscopy.

### Quantification of neutrophil-derived migrasomes and platelets at injury sites

The images of injury sites and in vitro flow channels were acquired by Dragonfly microscopy and processed using Imaris software. To count neutrophil-derived migrasomes, the surface analysis model was used to assess the number of Ly6G^+^ migrasomes at the injury site or the collagen-coated flow channel. To count platelets, the surface analysis model was used to assess and obtain the mean intensity (CD41) of each single platelet in the images. Then, the total intensity of CD41^+^ aggregated platelets at the injury site or collagen-coated flow channel was assessed and obtained. The total intensity was divided by the mean intensity to give the rough number of platelets.

### Isolation of NETs, NMPs and microvesicles

The isolation of NETs^13,14^ in vitro, NMPs in vitro^16,18^ and microvesicles in vivo^38^ was performed as previously described.

### FRET assay

Neutrophils were seeded on a fibronectin (10 μg ml^−1^)-coated surface in Hanks’ Balanced Salt Solution for 30 min at 37 °C under 5% CO_2_. Adherent cells, or purified neutrophil-derived migrasomes and platelets, were fixed with 4% paraformaldehyde for 15 min at room temperature and subjected to FRET assay. FRET was measured as previously described^30^.

### Statistics and reproducibility

Data analysis, statistical testing and visualization were conducted in GraphPad Prism 9. The figure captions describe the statistical approach used for each analysis. The results are presented as means ± s.e.m. when not otherwise indicated. A two-tailed unpaired Student’s *t*-test was applied for the comparison of two groups. Statistical differences are indicated as exact *P* values in the figures. The data distribution was assumed to be normal, but this was not formally tested. All statistical tests were performed at 95% confidence intervals. No statistical methods were used to predetermine sample size. Rather, sample size was determined based on similar studies in the field. The investigators were not blinded to allocation during the experiments and outcome assessment. Each biological experiment was obtained from different and independent samples. Quantification and statistics were derived from *n* = 3 independent experiments unless specified in the figure captions. All other experiments were performed a minimum of twice but usually three to four times independently. The experiments in Figs. 1b,g,h,k,m,o,q, 2c–e,i–k, 3d–g, 5a,d and 6a,c,d,g,j and Extended Data Figs. 1b,c,g–i,m–o and 2b were performed three times, whereas the experiments in Figs. 2g and 5f and Extended Data Figs. 1j–l, 2a and 6a–c,i were performed twice, with the representative results presented. For the in vivo studies, age- and sex-matched mice were randomly assigned to experimental and control groups for all of the experiments and the animal numbers used in the experiments are all indicated in the corresponding figure captions. No data were excluded from the analyses.

### Reporting summary

Further information on research design is available in the Nature Portfolio Reporting Summary linked to this article.

## Online content

Any methods, additional references, Nature Portfolio reporting summaries, source data, extended data, supplementary information, acknowledgements, peer review information; details of author contributions and competing interests; and statements of data and code availability are available at 10.1038/s41556-024-01440-9.

### Supplementary information

Supplementary Information Supplementary Figs. 1–3.

Reporting Summary

Supplementary Video 1 Circulating neutrophils generate a large number of neutrophil-derived migrasomes in blood. Three-dimensional time-lapse intravital imaging of neutrophils and neutrophil-derived migrasomes in mouse liver. PE–anti-Ly6G (Gr1) and AF647–WGA were injected (i.v.) for imaging using Dragonfly microscopy. Neutrophils and neutrophil-derived migrasomes are labelled with PE–anti-mouse Ly6G (green). AF647–WGA labels blood vessels (purple). Scale bar, 10 μm. Time interval, 60 s. Time duration, 1 h.

Supplementary Video 2 Human primary neutrophils generate a large number of migrasomes in vitro. Three-dimensional time-lapse imaging of human neutrophils in vitro. Neutrophils were isolated from human peripheral blood and seeded in a dish coated with fibronectin (10 μg ml^−1^; 30 min; 37 °C) for live-cell time-lapse imaging using the Dragonfly microscopy system. Neutrophils were labelled with APC–anti-CD16 and PE–anti-CD66b. Time interval and duration (h:min:s) are indicated in the video. Scale bar, 7 μm.

### Source data

Source Data Figs. 1–7 and Extended Data Figs. 4–7 A single file containing all of the numerical source data for Figs. 1–7 and Extended Data Figs. 4–7.

Source Data Figs. 1–7 and Extended Data Figs. 1–7 A single file containing all of the unprocessed blots and/or gels for Figs. 1–7 and Extended Data Figs. 1–7.

## Data Availability

The mass spectrometry proteomics data have been deposited to the ProteomeXchange Consortium via the PRIDE partner repository with the dataset identifiers PXD051229 (mouse neutrophil-derived migrasomes), PXD051231 (human neutrophil-derived migrasomes) and PXD051246 (*Tspan9*). The mass spectrometry lipidomics data have been deposited to the ProteomeXchange Consortium via the PRIDE partner repository with the dataset identifier PXD051238. RNA sequencing data that support the findings of this study have been deposited in the National Center for Biotechnology Information Sequence Read Archive under the accession codes PRJNA1097219 and SRR28578135. The database for mass spectrometry is from UniProt (https://www.uniprot.org/). Source data are provided with this paper. All other data supporting the findings of this study are available from the corresponding author on reasonable request.
