# Supplementary material for: Neutrophil-derived migrasomes are an essential part of the coagulation system
Source: Nat Cell Biol. 2024 Jul 12;26(7):1110–23. doi: 10.1038/s41556-024-01440-9 (PMC11251984; doi:10.1038/s41556-024-01440-9)

nature cell biology

Supplementary information https://doi.org/10.1038/s41556-024-01440-9

Neutrophil-derived migrasomes are an essential part of the coagulation system

In the format provided by the authors and unedited

Supplementary Fig. 1 | Representative gating strategies of flow cytometry analysis for all neu-migrasomes and NMPs in Fig. 1l, 1p and Extended Data Fig. 1f. The forward and sideward scatters are used to select the starting populations. Single particles are then selected using FSC-A and FSC-H, and then particles are sorted based on their fluorescent intensity in the Ly6G-PE and/or any other indicated fluorescent channel. Only the panels with PE and/or FITC intensity are shown in the figures of the manuscript.

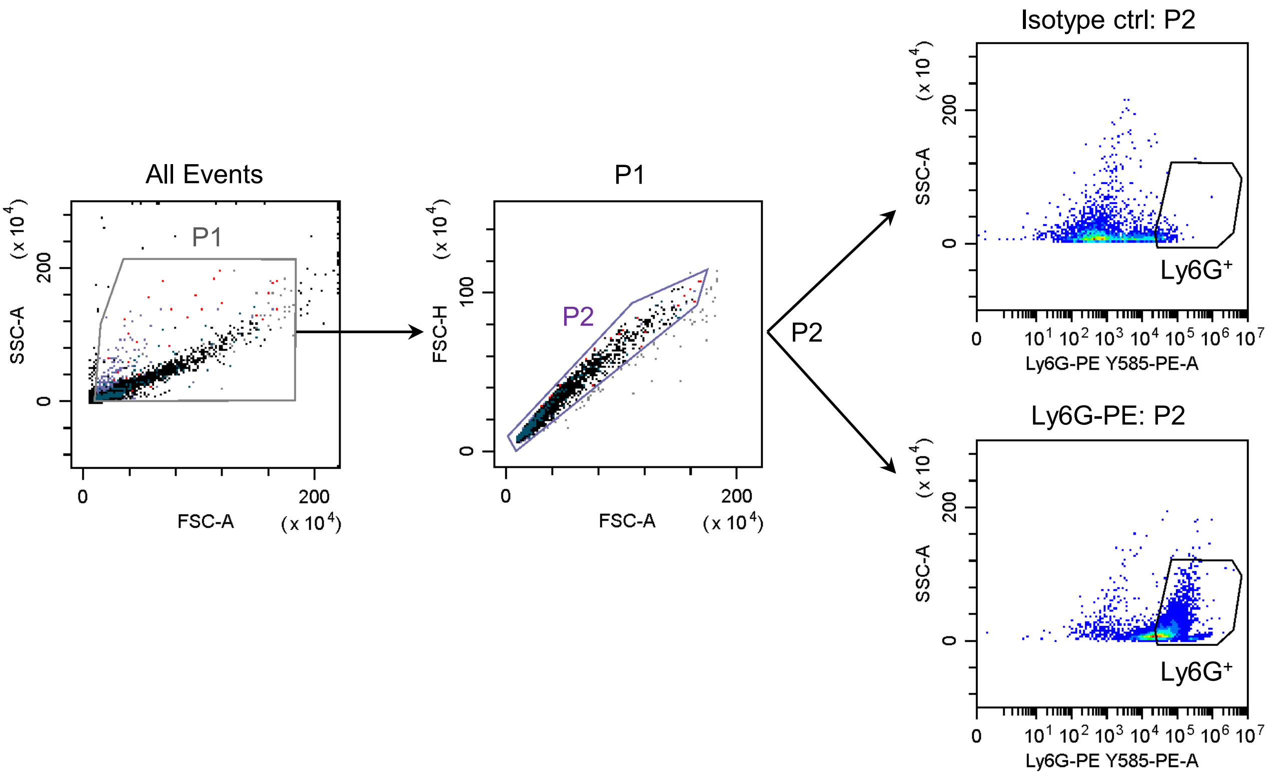

Supplementary Fig. 2 | Representative gating strategies of flow cytometry analysis for all platelets in Fig. 4a-b, g, 6h-i and Extended Data Fig. 3a. The forward and sideward scatters are used to select the starting populations. Single particles are then selected using FSC-A and FSC-H. Particles are sorted based on their fluorescent intensity in the CD41-APC, and then the particles are sorted based on their fluorescent intensity in the CD41-APC, CD62P-PE. Only the panels with PE and APC intensities are shown in the figures of the manuscript. The lower panel of the example shown here come from Fig. 4a.

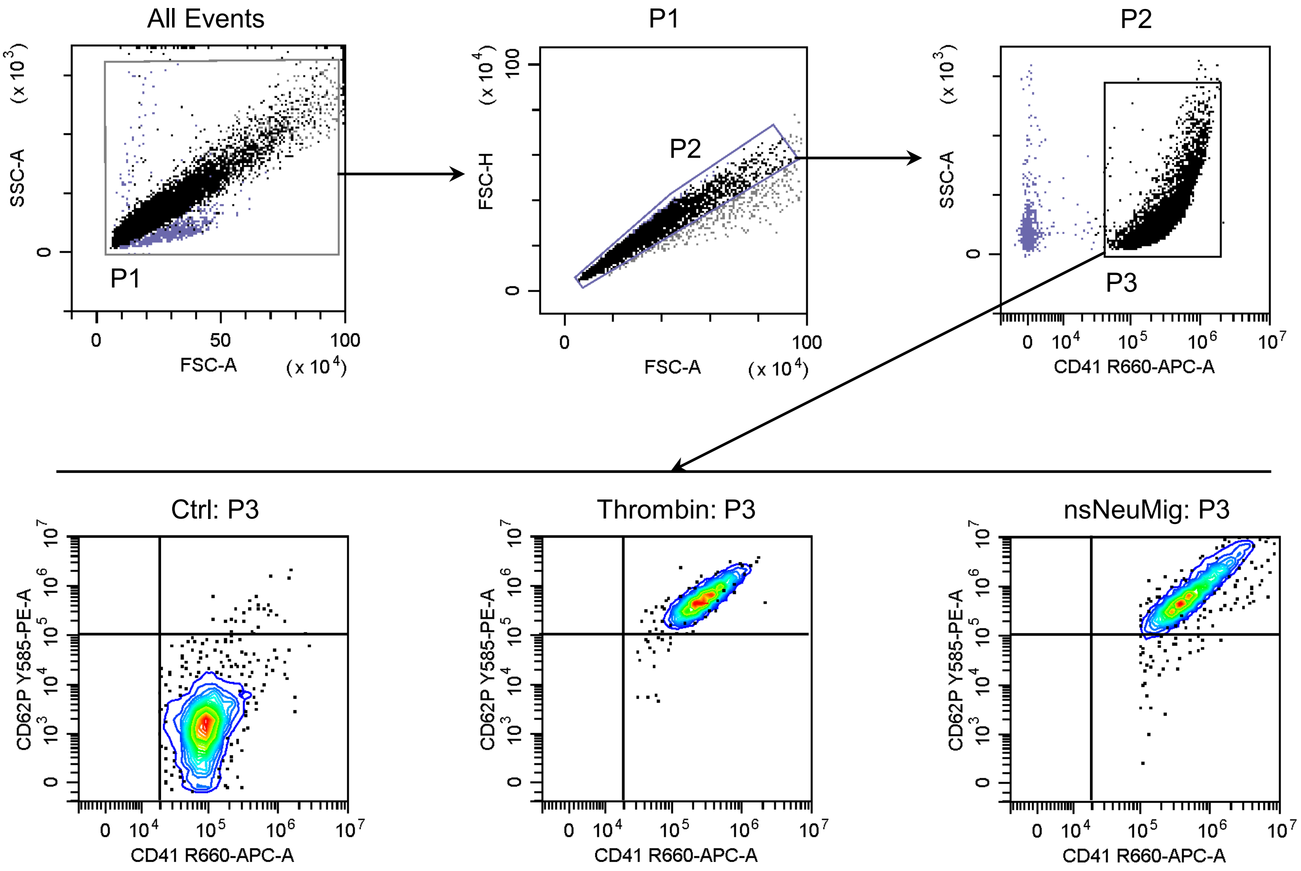

Supplementary Fig. 3 | Representative gating strategies of flow cytometry analysis for all blood cells in Extended Data Fig. 1a, e, 5a-b. The forward and sideward scatters are used to select the starting populations. Single particles are then selected using FSC-A and FSC-H. Particles are sorted based on their fluorescent intensity in the CD41-APC and Ly6G-PE. Only the panels with PE and/or APC intensities are shown in the figures of the manuscript. The right panel of the example shown here come from Extended Data Fig. 1a.

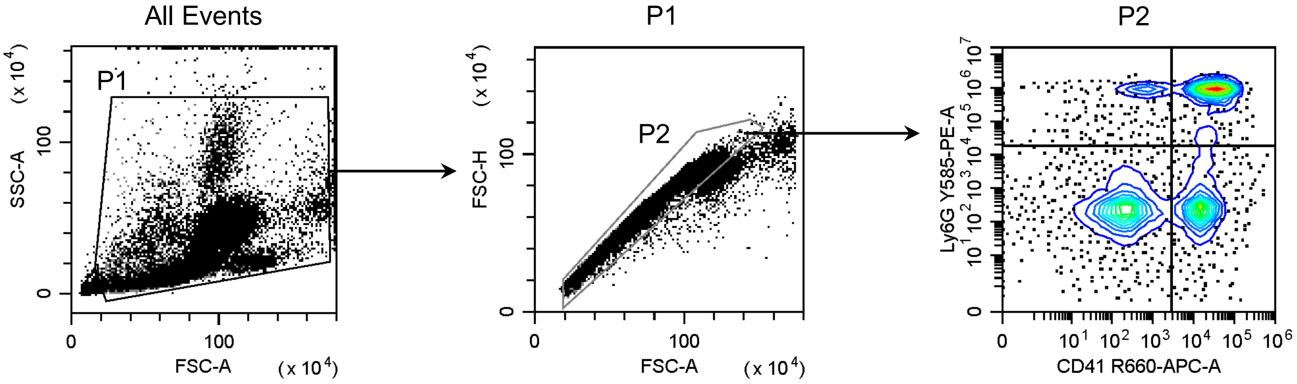

Supplement: Supplementary file 1 — Supplementary Figs. 1–3. [file 41556_2024_1440_MOESM1_ESM.pdf]
