# Supplementary material for: Neutrophil-derived migrasomes are an essential part of the coagulation system
Source: Nat Cell Biol. 2024 Jul 12;26(7):1110–23. doi: 10.1038/s41556-024-01440-9 (PMC11251984; doi:10.1038/s41556-024-01440-9)
Supplement: Supplementary file 2 — Reporting Summary [file 41556_2024_1440_MOESM2_ESM.pdf]

nature portfolio

Corresponding author(s): Li Yu

Last updated by author(s): May 8, 2024

Reporting Summary

Nature Portfolio wishes to improve the reproducibility of the work that we publish. This form provides structure for consistency and transparency in reporting. For further information on Nature Portfolio policies, see our Editorial Policies and the Editorial Policy Checklist.

Statistics

For all statistical analyses, confirm that the following items are present in the figure legend, table legend, main text, or Methods section.

n/a	Confirmed	
☐	☒	The exact sample size (*n*) for each experimental group/condition, given as a discrete number and unit of measurement
☐	☒	A statement on whether measurements were taken from distinct samples or whether the same sample was measured repeatedly
☐	☒	The statistical test(s) used AND whether they are one- or two-sided<br>*Only common tests should be described solely by name; describe more complex techniques in the Methods section.*
☒	☐	A description of all covariates tested
☐	☒	A description of any assumptions or corrections, such as tests of normality and adjustment for multiple comparisons
☐	☒	A full description of the statistical parameters including central tendency (e.g. means) or other basic estimates (e.g. regression coefficient) AND variation (e.g. standard deviation) or associated estimates of uncertainty (e.g. confidence intervals)
☐	☒	For null hypothesis testing, the test statistic (e.g. *F*, *t*, *r*) with confidence intervals, effect sizes, degrees of freedom and *P* value noted<br>*Give P values as exact values whenever suitable.*
☒	☐	For Bayesian analysis, information on the choice of priors and Markov chain Monte Carlo settings
☒	☐	For hierarchical and complex designs, identification of the appropriate level for tests and full reporting of outcomes
☒	☐	Estimates of effect sizes (e.g. Cohen's *d*, Pearson's *r*), indicating how they were calculated

Our web collection on statistics for biologists contains articles on many of the points above.

Software and code

Policy information about availability of computer code

Data collection	Imaging data were acquired by Dragonfly Fusion (Andor). Imaging-flow cytometry analysis were conducted with ImageStream MKII flow cytometer (Luminex) and the data were collected by Inspire software. Proteomics data were collected using UltiMateTM 3000 RSLC nano system. Lipidomics data were collected using a Q-Exactive HFX orbitrap mass spectrometer (Thermo Fisher, CA). Western blots were imaged on ChemiDoc MP Imaging System (BIO-RAD).
Data analysis	Image analysis was performed with Imaris 9.5.0 and Image J2. Statistical testing analysis was conducted by Graphpad Prism 9. Imaging-flow cytometry data were analyzed by IDEAS software (Luminex). DIA-MS data were analyzed using the Spectronaut 15.6 software. Lipidomics data analysis was performed by the software lipidsearch (Thermo Fisher, CA).

For manuscripts utilizing custom algorithms or software that are central to the research but not yet described in published literature, software must be made available to editors and reviewers. We strongly encourage code deposition in a community repository (e.g. GitHub). See the Nature Portfolio guidelines for submitting code & software for further information.

Data

Policy information about availability of data

All manuscripts must include a data availability statement. This statement should provide the following information, where applicable:

- Accession codes, unique identifiers, or web links for publicly available datasets
- A description of any restrictions on data availability
- For clinical datasets or third party data, please ensure that the statement adheres to our policy

The mass spectrometry proteomics data have been deposited to the ProteomeXchange Consortium via the PRIDE partner repository with the dataset identifier PXD051229 (mouse neu-migrasomes), PXD051231 (human neu-migrasomes), and PXD051246 (Tspan9). The mass spectrometry lipidomics data have been deposited to the ProteomeXchange Consortium via the PRIDE partner repository with the dataset identifier PXD051238. RNA sequencing data that support the findings of this study have been deposited in the National Center for Biotechnology Information Sequence Read Archive under the accession codes PRJNA1097219 and SRR28578135. The database for mass spectrometry is from uniprot (https://www.uniprot.org/). Source data have been provided in Source Data. All other data supporting the findings of this study are available from the corresponding author on reasonable request.

Research involving human participants, their data, or biological material

Policy information about studies with human participants or human data. See also policy information about sex, gender (identity/presentation), and sexual orientation and race, ethnicity and racism.

Reporting on sex and gender	Sex and gender were not considered in this study and the information was not collected as part of our protocol.
Reporting on race, ethnicity, or other socially relevant groupings	Race, ethnicity, or other socially relevant groupings were not considered in this study.
Population characteristics	In human related in vitro coagulation study, the human research participants are healthy volunteers, 18-60 years old. No matter what the gender is.
Recruitment	Human bloods were collected from healthy volunteers. There is no self-selection bias.
Ethics oversight	All human blood experiments were approved by the ethics committee on human research at the West China Hospital, Sichuan University, permission number 2023 Review (No.4), 2023 Review (No.1546) and the Institution Review Board of Tsinghua University, project number 20220193. Informed consent was obtained from the participants. No compensation was offered to any participant.

Note that full information on the approval of the study protocol must also be provided in the manuscript.

Field-specific reporting

Please select the one below that is the best fit for your research. If you are not sure, read the appropriate sections before making your selection.

☒ Life sciences ☐ Behavioural & social sciences ☐ Ecological, evolutionary & environmental sciences

For a reference copy of the document with all sections, see nature.com/documents/nr-reporting-summary-flat.pdf

Life sciences study design

All studies must disclose on these points even when the disclosure is negative.

Sample size	No statistical methods were used to predetermine sample sizes. Rather, sample size was determined based on similar studies in the field, and are based on the different experimental procedures e.g. technical difficulty, variation of experiments. The sample size for each experiment is included in the respective figure legend.
Data exclusions	No data were excluded from the analysis.
Replication	All experimental findings were replicated successfully. Numbers of attempts of replication are described in the figure legends.
Randomization	For in vivo experiments in mice, wild type animals were randomized before treatment. For experiments involved with knockout mice, the experimental and control groups mice were determined by genotyping and randomly selected from either sex for experiments. No randomization of samples was relevant to other experiments since they were internally controlled.
Blinding	The investigators were not blinded to allocation during experiments and outcome assessment as data were collected using unbiased methods, and data analyses were performed by software therefore in an unbiased manner, making blinding therefore not applicable.

Reporting for specific materials, systems and methods

We require information from authors about some types of materials, experimental systems and methods used in many studies. Here, indicate whether each material, system or method listed is relevant to your study. If you are not sure if a list item applies to your research, read the appropriate section before selecting a response.

Materials & experimental systems

n/a	Involved in the study
☐	☒ Antibodies
☒	☐ Eukaryotic cell lines
☒	☐ Palaeontology and archaeology
☐	☒ Animals and other organisms
☒	☐ Clinical data
☒	☐ Dual use research of concern
☒	☐ Plants

Methods

n/a	Involved in the study
☒	☐ ChIP-seq
☐	☒ Flow cytometry
☒	☐ MRI-based neuroimaging

Antibodies

Antibodies used

Imaging and flow cytometry antibodies:
PE anti-Mouse Ly6G (Gr1), eBioscience, cat# 12-5931-82
PE anti-mouse Ly6G, Biolegend, Cat# 127607
Alexa Fluor® 488 anti-mouse Ly6G, Biolegend, Cat# 127625
APC anti-mouse CD41, Biolegend, Cat# 133913
PE anti-mouse CD41, Biolegend, Cat# 133905
PE anti-mouse/rat CD62P (P-selectin), Biolegend, Cat# 148305
Alexa Fluor® 647 anti-Ly6B.2, Bio-Rad, Cat# MCA771A647T
FITC anti-Ly6B.2, Bio-Rad, Cat# MCA771FT
FITC anti-mouse/rat CD61, Biolegend, Cat# 104305
FITC anti-Hu CD41a, BD-PMG, Cat# 555466
APC anti-CD62P, BD-PMG, Cat# 550888
Alexa Fluor® 647 anti-Hu CD66b, BD-PMG, Cat# 561645
PE anti- Ms IgG2a Kpa ItCl, BD-PMG, Cat# 555574
Alexa Fluor® 647 anti- Ms IgM KPA ItCl, BD-PMG, Cat# 560806
APC anti-human CD41, Biolegend, Cat# 303709
FITC anti-human CD235a (Glycophorin A), Biolegend, Cat# 349103
PE anti-human CD62P (P-Selectin), Biolegend, Cat# 304905
Alexa Fluor® 488 anti-human CD16, Biolegend, Cat# 302022
PE anti-human CD66b, Biolegend, Cat# 392903
Pacific Blue™ anti-human CD235a (Glycophorin A), Biolegend, Cat# 349108
Brilliant Violet 510™ anti-mouse TER-119, Biolegend, Cat# 116237
FITC anti-human CD62P (P-Selectin), Biolegend, Cat# 304903
PE anti-human CD41, Biolegend, Cat# 303705
Goat anti-Rat IgG (H+L)-Alexa Fluor™ 488, ThermoFisher, Cat# A-11006
Goat anti-Rabbit IgG (H+L)-Alexa Fluor™ 546, ThermoFisher, Cat# A-11010
Anti-Mouse CD29, BD Bioscience, Cat# 553715
Anti-Integrin β1, EMD Millipore, Cat# MAB2079Z
Anti-HA, CST, Cat# 3724S
Anti-AIIB2 is a kind gift from Jianfeng Chen lab.

Depletion antibodies:
Anti-mouse Ly6G, BioXCell, Cat# BP0075-1
Anti-mouse CD41, BD Bioscience, Cat# 553847
Rat IgG2a isotype control, BioXCell, Cat# BP0089
Rat IgG2b isotype control , BioXcell, Cat# BE0090

Western blot antibodies:
Anti-thrombin, GeneTex, GTX101270
Anti-prothrombin, Abcam, Cat# ab208590
Anti-Coagulation Factor X, Novus, NBP1-33320
Anti-Factor XIII, Abcam, Cat# ab185215
Anti-Factor VIII, Abcam, Cat# ab275376
Anti-Von Willebrand Factor, Abcam, Cat# ab174290
Anti-Factor XI, GeneTex, Cat# GTX113690-S
Anti-Factor XII, Abcam, Cat# ab196670
Anti-Fibrinogen alpha chain, Abcam, Cat# ab92572
Anti-Ly6G, CST, Cat# 87048S
Anti-Myeloperoxidase, R&D, Cat# AF3667-SP
Anti-CD41, Abcam, Cat# ab181582

Anti-β-Actin, Zen BioScience, Cat# 200068-8F10
Anti-GAPDH, Proteintech, Cat# 60004-1-Ig
Anti-Histone H3 (citrulline R2 + R8 + R17), Abcam, Cat# ab5103
Anti-Integrin α5, Cellsignal, Cat# 4705
Anti-CPQ, ABclonal, Cat# A12062
Anti-NDST, Santa Cruz, Cat# sc-374529
Anti-Tim23, BD Bioscience, Cat# 611222
Anti-CD41, Abcam, Cat# ab134131
Anti-Prothrombin, Abcam, Cat# ab208589
Anti-CD16, Abcam, Cat# ab246222
Anti-CD66b, Abcam, Cat# ab170875
Anti-CD11b, Abcam, Cat# ab184308
Anti-Integrin alpha 1, Abcam, Cat# ab181434
Anti-Integrin alpha 2, Abcam, Cat# ab133557
Anti-Integrin beta 1, Abcam, Cat# ab179471
Anti-Integrin beta 2, CST, Cat# 72607S
Anti-Integrin alpha 4, CST, Cat# 8440T
Anti-CD11a/Integrin αL, Abcam, Cat# ab228964
Anti-GLG1/ESL-1, Abcam, Cat# ab271182
Anti-CD44, Abcam, Cat# ab189524
Anti-PSGL-1/CD162, Novus, Cat# NB100-78039SS
Anti-CD62L, Abcam, Cat# ab253240
Anti-CD9, Abcam, Cat# ab92726
Anti-ALIX, Abcam, Cat# ab275377
Anti-CD63, Abcam, Cat# ab217345
Anti-KIF23, Proteintech, Cat# 28587-1-AP
Anti-ARF6, Abcam, Cat# ab131261
Anti-AER61, Abcam, Cat# ab190693
Anti-PIGK, Abcam, Cat# ab201693
Anti-Factor V, Proteintech, Cat# 20963-1-AP
Anti-Factor IX, Proteintech, Cat# 21481-1-AP
Anti-TOM20, Abcam, Cat# ab283317
Anti-TRFL, Thermo Fisher, Cat# PA5-95513
Anti-MMP9, Abcam, Cat# ab76003
Anti-TAPA1/CD81, HUABIO, Cat# ET1611-87
Rabbit Anti-Goat IgG(H+L)-HRP, SouthernBiotech, Cat# 6160-05
Goat Anti-Rat IgM mu chain (HRP), Abcam, Cat# ab98373
Goat Anti-Rabbit IgG (H+L), Jackson, Cat# 111-035-003
Goat Anti-Mouse IgG (H+L), Jackson, Cat# 115-035-003
Goat Anti-Rat IgG (H+L), Jackson, Cat# 112-035-003
Goat Anti-Mouse IgG(H+L), Proteintech, Cat# SA00001-1

Isolation antibodies:
Biotin anti-mouse TER-119/Erythroid Cells, Biolegend, Cat# 116204
Biotin anti-mouse CD41, Biolegend, Cat# 133930
Biotin anti-human CD41, Biolegend, Cat# 303734
Biotin anti-human CD235ab, Biolegend, Cat# 306618
Biotin anti-CD41, Abcam, Cat# ab28065
Blocking antibody:
Anti-mouse CD49b, Biolegend, Cat# 103501

For flow cytometry analysis, all antibodies are diluted at 1:200 unless otherwise specified in methods or legends. For western blot, all antibodies are diluted at 1:2000 (primary) and 1:5000 (secondary) unless otherwise specified in methods or legends. For immunofluorescence staining, all antibodies are diluted at 1:200 (primary) and 1:500 (secondary) unless otherwise specified in methods or legends.

Validation

Antibody validation information can be found on manufacturers' website:
PE anti-Mouse Ly6G (Gr1): https://www.thermofisher.cn/cn/zh/antibody/product/Ly-6G-Ly-6C-Antibody-clone-RB6-8C5-Monoclonal/12-5931-82
PE anti-mouse Ly6G: https://www.biolegend.com/en-gb/products/pe-anti-mouse-ly-6g-antibody-4777?GroupID=BLG5803
Alexa Fluor® 488 anti-mouse Ly6G: https://www.biolegend.com/en-gb/product-preview/alexa-fluor-488-anti-mouse-ly-6g-antibody-7085
APC anti-mouse CD41: https://www.biolegend.com/en-gb/products/apc-anti-mouse-cd41-antibody-7592?GroupID=BLG10424
PE anti-mouse CD41: https://www.biolegend.com/en-gb/products/pe-anti-mouse-cd41-antibody-5897?GroupID=BLG10424
PE anti-mouse/rat CD62P (P-selectin): https://www.biolegend.com/en-gb/products/pe-anti-mouse-rat-cd62p-p-selectin-antibody-10806?GroupID=BLG15664
Alexa Fluor® 647 anti-Ly6B.2: https://www.bio-rad-antibodies.com/monoclonal/mouse-ly-6b-2-alloantigen-antibody-7-4-mca771.html?f=alexa%20fluor%C2%AE%20647
FITC anti-Ly6B.2: https://www.bio-rad-antibodies.com/monoclonal/mouse-ly-6b-2-alloantigen-antibody-7-4-mca771.html?f=fitc
FITC anti-mouse/rat CD61: https://www.biolegend.com/en-gb/products/fitc-anti-mouse-rat-cd61-antibody-79?GroupID=BLG270
FITC anti-Hu CD41a: https://www.bdbiosciences.com/zh-cn/products/reagents/flow-cytometry-reagents/research-reagents/single-color-antibodies-ruo/fitc-mouse-anti-human-cd41a.555466
APC anti-CD62P: https://www.bdbiosciences.com/en-us/products/reagents/flow-cytometry-reagents/research-reagents/single-color-antibodies-ruo/apc-mouse-anti-human-cd62p.550888
Alexa Fluor® 647 anti-Hu CD66b: https://www.bdbiosciences.com/zh-cn/products/reagents/flow-cytometry-reagents/research-reagents/single-color-antibodies-ruo/alexa-fluor-647-mouse-anti-human-cd66b.561645

PE anti- Ms IgG2a Kpa ItCl: https://www.bdbiosciences.com/en-us/products/reagents/flow-cytometry-reagents/research-reagents/flow-cytometry-controls-and-lysates/pe-mouse-igg2a-isotype-control.555574
Alexa Fluor® 647 anti- Ms IgM KPA ItCl: https://www.bdbiosciences.com/en-us/products/reagents/flow-cytometry-reagents/research-reagents/flow-cytometry-controls-and-lysates/alexa-fluor-647-mouse-igm-isotype-control.560806
APC anti-human CD41: https://www.biolegend.com/en-gb/sean-tuckers-tests/apc-anti-human-cd41-antibody-735
FITC anti-human CD235a (Glycophorin A): https://www.biolegend.com/en-gb/explore-new-products/fitc-anti-human-cd235a-glycophorin-a-antibody-6701?GroupID=BLG8825
PE anti-human CD62P (P-Selectin): https://www.biolegend.com/en-gb/products/pe-anti-human-cd62p-p-selectin-antibody-595
Alexa Fluor® 488 anti-human CD16: https://www.biolegend.com/en-gb/products/alexa-fluor-488-anti-human-cd16-antibody-2734
PE anti-human CD66b: https://www.biolegend.com/en-gb/products/pe-anti-human-cd66b-antibody-16302
Pacific Blue™ anti-human CD235a (Glycophorin A): https://www.biolegend.com/en-gb/products/pacific-blue-anti-human-cd235a-glycophorin-a-antibody-7880
Brilliant Violet 510™ anti-mouse TER-119: https://www.biolegend.com/en-gb/products/brilliant-violet-510-anti-mouse-ter-119-erythroid-cells-antibody-8243
FITC anti-human CD62P (P-Selectin): https://www.biolegend.com/en-gb/products/fitc-anti-human-cd62p-p-selectin-antibody-593
PE anti-human CD41: https://www.biolegend.com/en-gb/products/pe-anti-human-cd41-antibody-737
Goat anti-Rat IgG (H+L)-Alexa Fluor™ 488: https://www.thermofisher.cn/cn/zh/antibody/product/Goat-anti-Rat-IgG-H-L-Cross-Adsorbed-Secondary-Antibody-Polyclonal/A-11006
Goat anti-Rabbit IgG (H+L)-Alexa Fluor™ 546: https://www.thermofisher.cn/cn/zh/antibody/product/Goat-anti-Rabbit-IgG-H-L-Cross-Adsorbed-Secondary-Antibody-Polyclonal/A-11010
Anti-Mouse CD29: https://www.bdbiosciences.com/zh-cn/products/reagents/flow-cytometry-reagents/research-reagents/single-color-antibodies-ruo/purified-rat-anti-mouse-cd29.553715
Anti-Integrin β1: https://www.merckmillipore.com/CN/zh/product/Anti-Integrin-1-Antibody-activated-clone-HUTS-4-Azide-Free,MM_NF-MAB2079Z?ReferrerURL=https%3A%2F%2Fcn.bing.com%2F&bd=1
Anti-HA: https://www.cellsignal.com/products/primary-antibodies/ha-tag-c29f4-rabbit-mab/3724
Anti-AIIB2 is a kind gift from Jianfeng Chen lab, and validated in PMID: 26994136.
Anti-mouse Ly6G: https://bioxcell.com/invivoplus-anti-mouse-ly6g-bp0075-1
Anti-Mouse CD41: https://www.bdbiosciences.com/en-us/products/reagents/flow-cytometry-reagents/research-reagents/single-color-antibodies-ruo/purified-rat-anti-mouse-cd41.553847
Rat IgG2a isotype control: https://bioxcell.com/invivoplus-rat-igg2a-isotype-control-anti-trinitrophenol-bp0089
Rat IgG2b isotype control: https://bioxcell.com/invivomab-rat-igg2b-isotype-control-anti-keyhole-limpet-hemocyanin-be0090
Anti-prothrombin: https://www.abcam.cn/products/primary-antibodies/prothrombin-antibody-epr20131-ab208590.html
Anti-Coagulation Factor X: https://www.novusbio.com/products/coagulation-factor-x-antibody_nbp1-33320
Anti-Factor XIII: https://www.abcam.cn/products/primary-antibodies/factor-xiii-antibody-epr109862-ab185215.html
Anti-Factor VIII: https://www.abcam.cn/products/primary-antibodies/factor-viii-antibody-epr24039-262-ab275376.html
Anti-Von Willebrand Factor: https://www.abcam.cn/products/primary-antibodies/von-willebrand-factor-antibody-epr12011-ab174290.html
Anti-Factor XI: https://www.genetex.cn/Product/Detail/Factor-XI-antibody-C1C3/GTX113690
Anti-Factor XII: https://www.abcam.cn/products/primary-antibodies/factor-xii-antibody-c-terminal-ab196670.html
Anti-Fibrinogen alpha chain: https://www.abcam.com/products/primary-antibodies/fibrinogen-alpha-chain-antibody-epr2919-ab92572.html
Anti-Ly6G: https://www.cellsignal.com/products/primary-antibodies/ly-6g-e6z1t-rabbit-mab/87048
Anti-Myeloperoxidase: https://www.rndsystems.com/cn/products/human-mouse-myeloperoxidase-mpo-antibody_af3667
Anti-CD41: https://www.abcam.cn/products/primary-antibodies/cd41-antibody-epr17876-ab181582.html
Anti-β-Actin: http://www.zen-bioscience.com/prod_view.aspx?IsActiveTarget=True&TypeId=160&Id=421253&FId=t3:160:3
Anti-GAPDH: https://www.ptglab.co.jp/products/GAPDH-Antibody-60004-1-Ig.htm
Anti-Histone H3 (citrulline R2 + R8 + R17): https://www.abcam.cn/products/primary-antibodies/histone-h3-citrulline-r2--r8--r17-antibody-ab5103.html
Anti-Integrin α5: https://www.cellsignal.cn/products/primary-antibodies/integrin-a5-antibody/4705
Anti-CPQ: https://abclonal.com.cn/catalog/A12062
Anti-NDST: https://www.scbt.com/zh/p/ndst-antibody-e-9
Anti-Tim23: https://www.bdbiosciences.com/zh-cn/products/reagents/microscopy-imaging-reagents/immunofluorescence-reagents/purified-mouse-anti-tim23.611222
Anti-CD41: https://www.abcam.cn/products/primary-antibodies/cd41-antibody-epr4330-ab134131.html
Anti-Prothrombin: https://www.abcam.cn/products/primary-antibodies/prothrombin-antibody-epr20159-ab208589.html
Anti-CD16: https://www.abcam.cn/products/primary-antibodies/cd16-antibody-epr22409-124-ab246222.html
Anti-CD66b: https://www.abcam.cn/products/primary-antibodies/cd66b-antibody-epr7701-ab170875.html
Anti-CD11b: https://www.abcam.cn/products/primary-antibodies/cd11b-antibody-epr19387-ab184308.html
Anti-Integrin alpha 1: https://www.abcam.cn/products/primary-antibodies/integrin-alpha-1-antibody-c-terminal-ab181434.html
Anti-Integrin alpha 2: https://www.abcam.cn/products/primary-antibodies/integrin-alpha-2-antibody-epr5788-ab133557.html
Anti-Integrin beta 1: https://www.abcam.cn/products/primary-antibodies/integrin-beta-1-antibody-epr16895-ab179471.html
Anti-Integrin beta 2: https://www.cellsignal.jp/products/primary-antibodies/integrin-b2-e9o7w-rabbit-mab/72607
Anti-Integrin alpha 4: https://www.cellsignal.jp/products/primary-antibodies/integrin-a4-d2e1-xp-rabbit-mab/8440
Anti-CD11a/Integrin αL: https://www.abcam.cn/products/primary-antibodies/cd11a-antibody-epr22578-312-ab228964.html
Anti-GLG1/ESL-1: https://www.abcam.cn/products/primary-antibodies/glg1-antibody-epr24347-15-ab271182.html
Anti-CD44: https://www.abcam.cn/products/primary-antibodies/cd44-antibody-epr18668-ab189524.html
Anti-PSGL-1/CD162: https://www.novusbio.com/products/psgl-1-cd162-antibody-heca-452_nb100-78039
Anti-CD62L: https://www.abcam.cn/products/primary-antibodies/cd62l-antibody-epr17012-27-ab253240.html
Anti-CD9: https://www.abcam.com/products/primary-antibodies/cd9-antibody-epr2949-ab92726.html
Anti-ALIX: https://www.abcam.com/products/primary-antibodies/alix-antibody-epr23653-32-ab275377.html
Anti-CD63: https://www.abcam.com/products/primary-antibodies/cd63-antibody-epr21151-ab217345.html
Anti-KIF23: https://www.ptglab.co.jp/products/KIF23-Antibody-28587-1-AP.htm
Anti-ARF6: https://www.abcam.cn/products/primary-antibodies/arf6-antibody-epr8357-ab131261.html
Anti-AER61: https://www.abcam.cn/products/primary-antibodies/aer61-antibody-epr12944-ab190693.html
Anti-PIGK: https://www.abcam.cn/products/primary-antibodies/pigk-antibody-epr17843-ab201693.html
Anti-Factor V: https://www.ptglab.co.jp/Products/F5-Antibody-20963-1-AP.htm

Anti-Factor IX: https://www.ptglab.co.jp/Products/F9-Antibody-21481-1-AP.htm
Anti-TOM20: https://www.abcam.cn/products/primary-antibodies/tomm20-antibody-epr15581-39-mouse-igg1-chimeric-ab283317.html
Anti-TRFL: https://www.thermofisher.cn/cn/zh/antibody/product/Lactoferrin-Antibody-Polyclonal/PA5-95513
Anti-MMP9: https://www.abcam.cn/products/primary-antibodies/mmp9-antibody-ep1254-ab76003.html
Anti-TAPA1/CD81: http://www.huabio.cn/products/TAPA1-CD81-antibody-ET1611-87
Rabbit Anti-Goat IgG(H+L)-HRP: https://www.southernbiotech.com/rabbit-anti-goat-igg-h-l-hrp-6160-05
Goat Anti-Rat IgM mu chain (HRP): https://www.abcam.com/products/secondary-antibodies/goat-rat-igm-mu-chain-hrp-preadsorbed-ab98373.html
Goat Anti-Rabbit IgG (H+L): https://www.jacksonimmuno.com/catalog/products/111-035-003
Goat Anti-Mouse IgG (H+L): https://www.jacksonimmuno.com/catalog/products/115-035-003
Goat Anti-Rat IgG (H+L): https://www.jacksonimmuno.com/catalog/products/112-035-003
Goat Anti-Mouse IgG(H+L): https://www.ptglab.co.jp/products/HRP-conjugated-Affinipure-Goat-Anti-Mouse-IgG-H-L-secondary-antibody.htm
Biotin anti-mouse TER-119/Erythroid Cells: https://www.biolegend.com/en-gb/sean-tuckers-tests/biotin-anti-mouse-ter-119-erythroid-cells-antibody-1864?GroupID=ImportedGROUP1
Biotin anti-mouse CD41: https://www.biolegend.com/en-gb/products/biotin-anti-mouse-cd41-antibody-13059
Biotin anti-human CD41: https://www.biolegend.com/en-gb/products/biotin-anti-human-cd41-antibody-14577
Biotin anti-human CD235ab: https://www.biolegend.com/en-us/products/biotin-anti-human-cd235ab-antibody-12031
Biotin anti-CD41: https://www.abcam.cn/products/primary-antibodies/biotin-cd41-antibody-mem-06-ab28065.html
Anti-mouse CD49b: https://www.biolegend.com/de-de/products/purified-anti-mouse-cd49b-antibody-300?GroupID=BLG4895

Anti-Thrombin antibody was validated for the species by western blot as shown in Fig. 3 (mouse), 6 (human). Depletion antibodies, including anti-mouse Ly6G and anti-mouse CD41, were validated by the manufacturers which can be found on the website and also validated by flow cytometry as shown in ED Fig. 5a-b. All of the other antibodies were validated by the manufacturer and the antibody validation information can be found on manufacturers' website.

Animals and other research organisms

Policy information about studies involving animals; ARRIVE guidelines recommended for reporting animal research, and Sex and Gender in Research

Laboratory animals	C57BL/6J were purchased from animal center of Tsinghua University. Tspan9 KO and cKO mice were generated from GemPharmatech Co. Ltd. LysM-Cre mice were obtained from Dr. Xiaoyu Hu's lab in Tsinghua University. Tspan9-HA mice were generated by the Genome Tagging Project (GTP) at the Shanghai Institute of Biochemistry and Cell Biology (SIBCB). 6-12 weeeks old male or female mice were used in the experiments. Mice were housed in ventilated cages in a specific pathogen-free animal facility under a 12 hr light/12 hr dark cycle with 20–26°C, 40–70% humidity.
Wild animals	None of the wild animals used.
Reporting on sex	This study did not involve sex based analyses.
Field-collected samples	This study did not involve samples collected from field.
Ethics oversight	All animal experiments were approved by the Institutional Animal Care and Use Committee and conducted in accordance of governmental and Tsinghua guidelines for animal welfare.

Note that full information on the approval of the study protocol must also be provided in the manuscript.

Plants

Seed stocks	No plants used.
Novel plant genotypes	No plants used.
Authentication	No plants used.

Flow Cytometry

Plots

Confirm that:

☒ The axis labels state the marker and fluorochrome used (e.g. CD4-FITC).

☒ The axis scales are clearly visible. Include numbers along axes only for bottom left plot of group (a 'group' is an analysis of identical markers).

☒ All plots are contour plots with outliers or pseudocolor plots.

☒ A numerical value for number of cells or percentage (with statistics) is provided.

Methodology

Sample preparation	For blood cell analysis and sorting, mouse blood was collected from the ocular venous plexus and put into a tube containing blood collection buffer (PBS supplemented with 20 mM EDTA on ice). The blood mixture was then centrifuged at 800 g, 4℃, 5 min, and the cell pellet was resuspended with Ammonium-Chloride-Potassium (ACK) lysis buffer for 2 min to lyse red blood cells. The lysate was then centrifuged at 1000 g, 4℃, 5 min and the supernatant was removed. The pellet was resuspended with PBS and stained with PE anti-Ly6G and APC anti-CD41 at room temperature for 15 min and then centrifuged at 1000 g, 4℃, 5 min to obtain the blood cell mixture. The cell mixture was resuspended with PBS for flow cytometry sorting by MoFlo Astrios EQ (Beckman Coulter) or MoFlo XDP (Beckman Coulter) and imaging by Dragonfly spinning disk microscopy (Andor). For blood migrasome analysis, blood migrasomes were purified from mouse blood and stained with AF647 anti-Ly-6G at room temperature for 15 min. AF647 Rat IgG2a served as a staining control. The migrasome mixture was centrifuged at 20000 g, 4℃, 30 min. The migrasome pellet was resuspended with PBS for flow cytometry analysis using a CytoFlex LX (Beckman Coulter).
Instrument	Data were analyzed on CytoFlex (Beckman Coulter); Samples were sorted by MoFlo Astrios EQ (Beckman Coulter); Imaging-flow cytometry analysis conducted with ImageStream MKII flow cytometer (Luminex).
Software	CytExpert software (Beckman Coulter) was used to analyze the data on CytoFlex; Summit software was used to analyze the data collected from MoFlo Astrios EQ (Beckman Coulter); IDEAS software (Luminex) was used to analyze the data collected from ImageStream MKII flow cytometer (Luminex).
Cell population abundance	For the cell population, a customized gate was draw based on the control samples and applied to all other samples to obtain a population abundance for each samples.
Gating strategy	Single particles were gated on FSC/SSC, followed by gating a customized gate based on the control samples and applied to all other samples to obtain a population abundance for each samples.

☒ Tick this box to confirm that a figure exemplifying the gating strategy is provided in the Supplementary Information.
