# Supplementary material for: Neutrophil-derived migrasomes are an essential part of the coagulation system
Source: Nat Cell Biol. 2024 Jul 12;26(7):1110–23. doi: 10.1038/s41556-024-01440-9 (PMC11251984; doi:10.1038/s41556-024-01440-9)

Unprocessed Blot Images

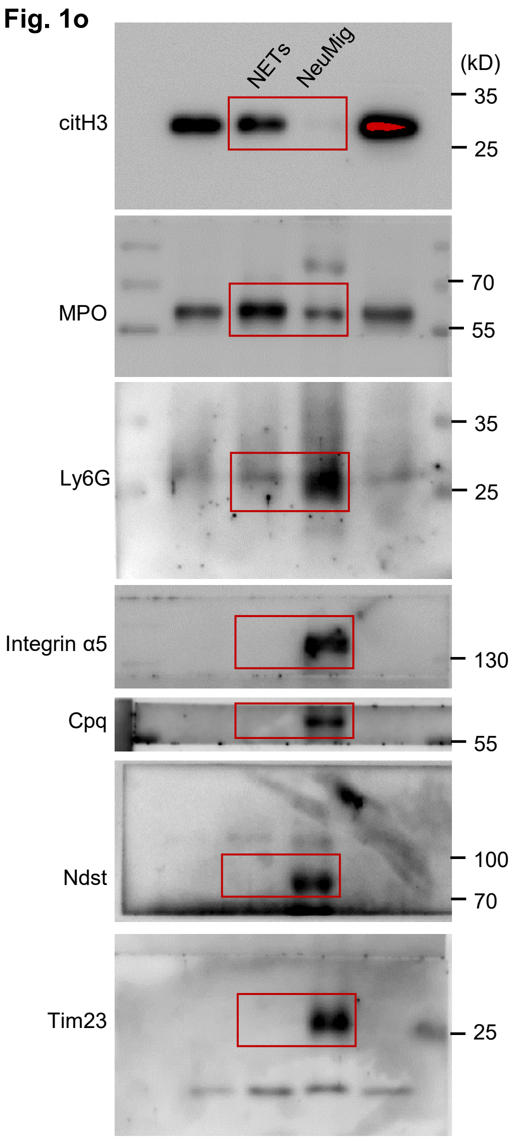

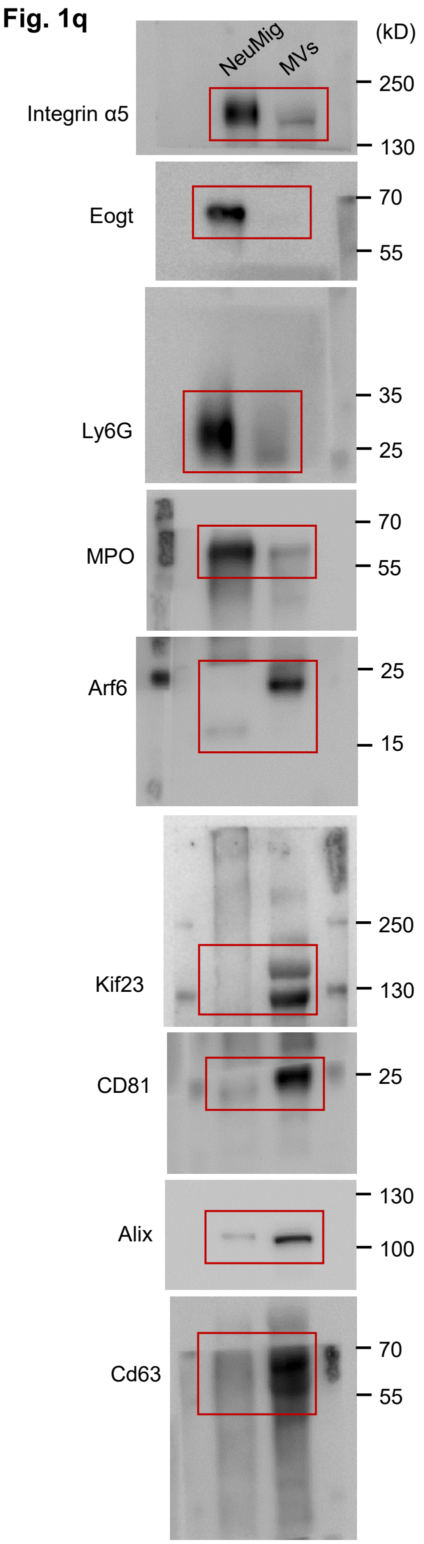

Fig. 2c

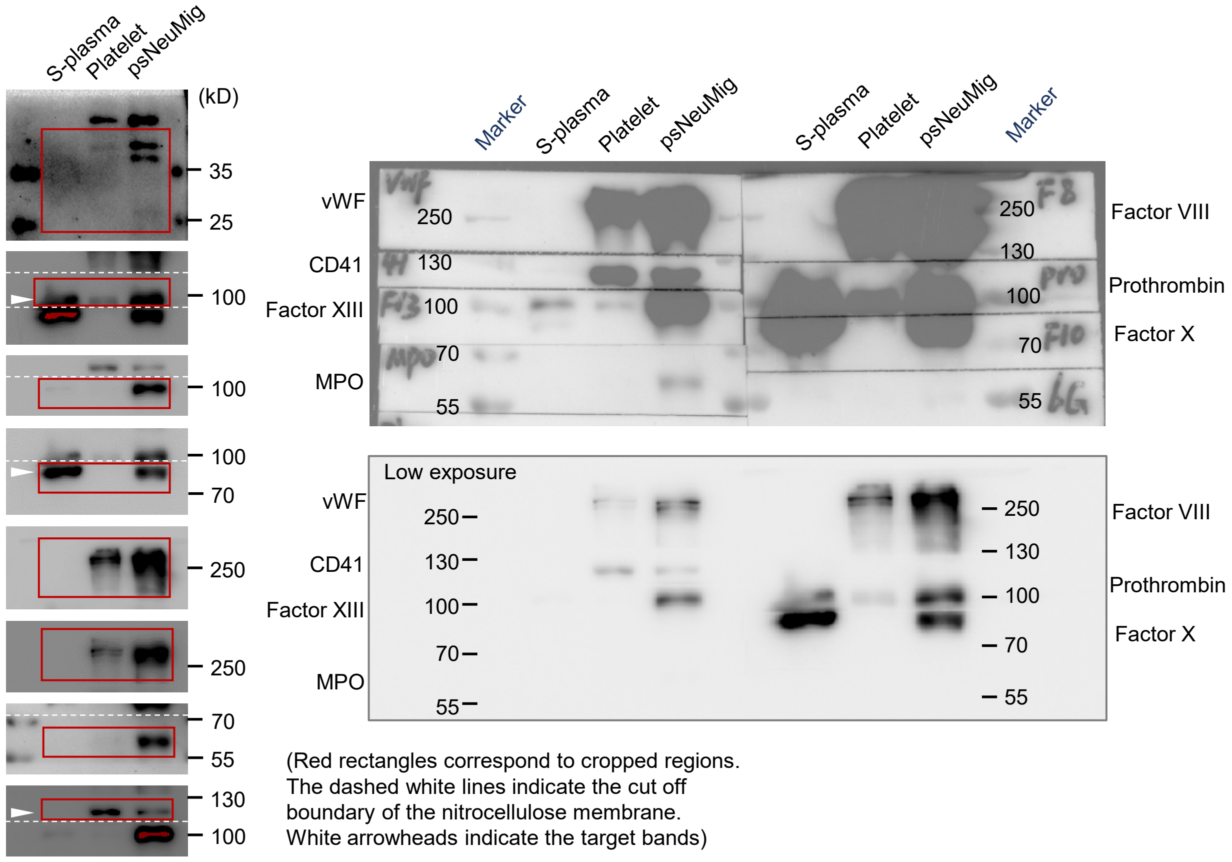

(Red rectangles correspond to cropped regions.
The dashed white lines indicate the cut off
boundary of the nitrocellulose membrane.
White arrowheads indicate the target bands)

Fig. 2g

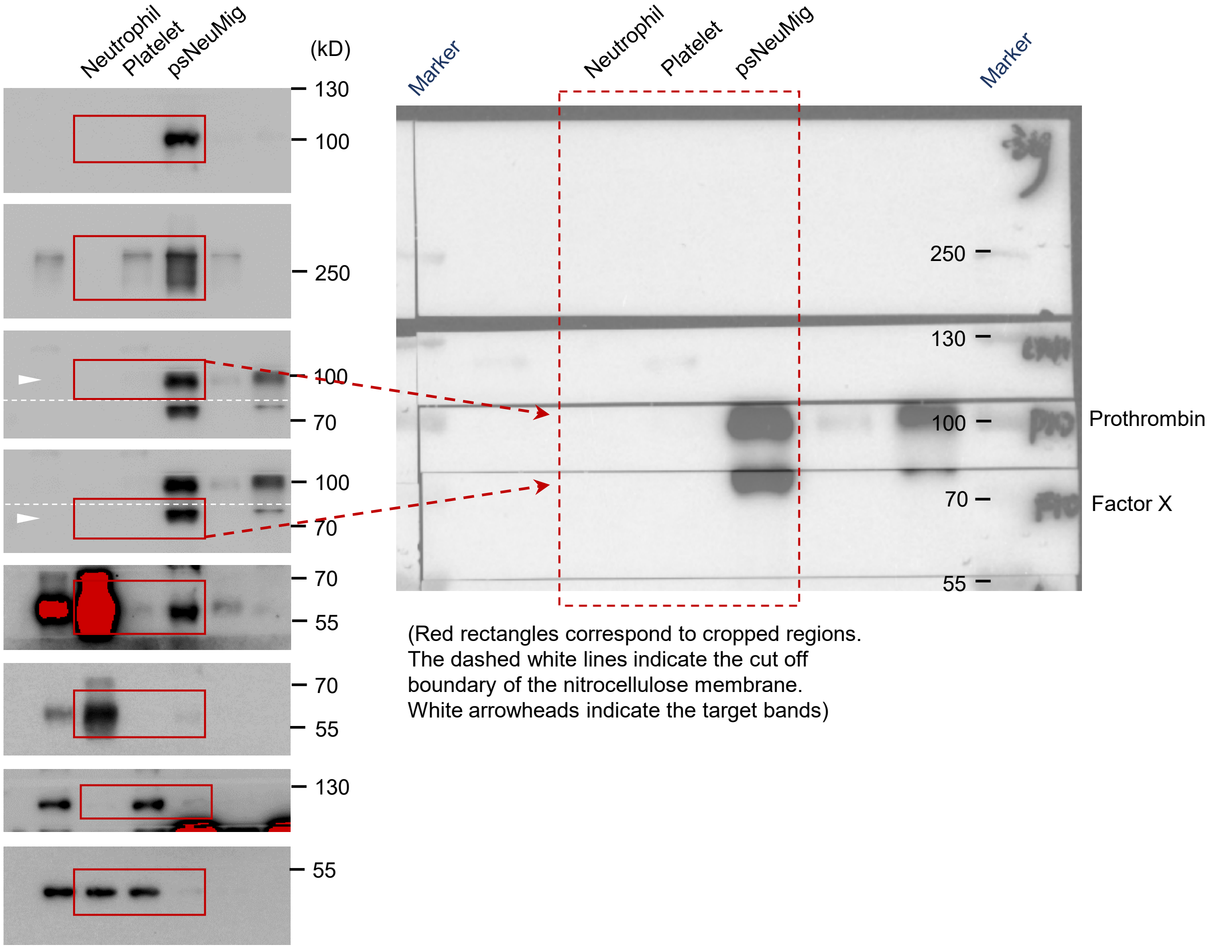

(Red rectangles correspond to cropped regions.
The dashed white lines indicate the cut off
boundary of the nitrocellulose membrane.
White arrowheads indicate the target bands)

Fig. 2d

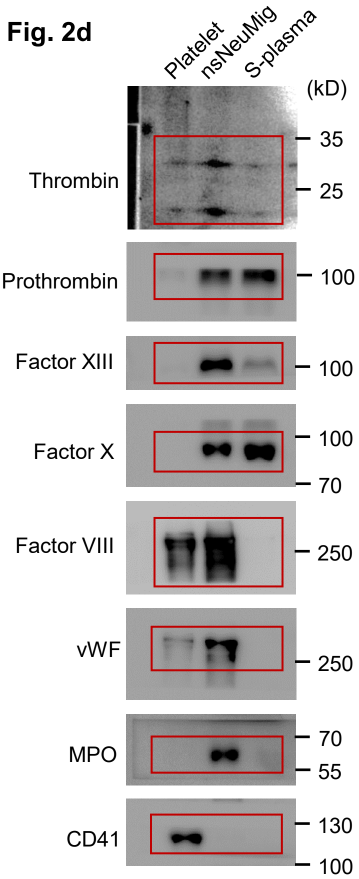

Fig. 2i

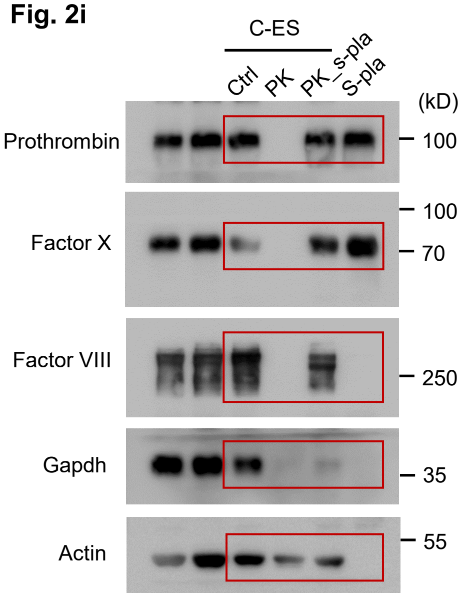

Fig. 2j

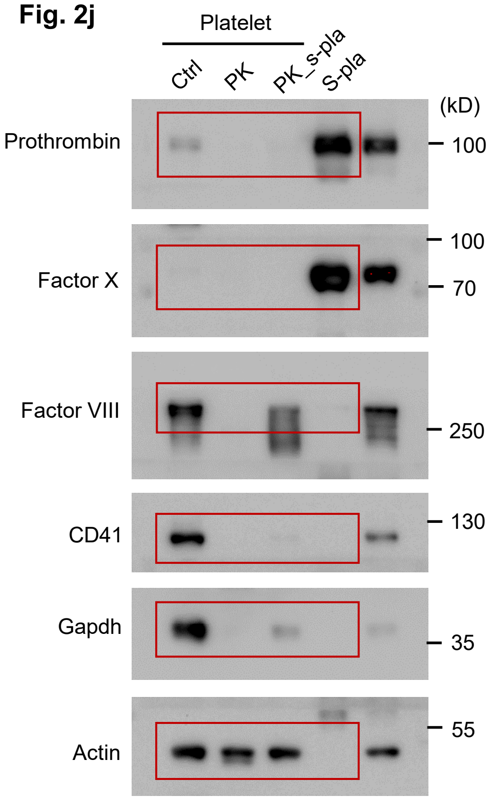

Fig. 2k

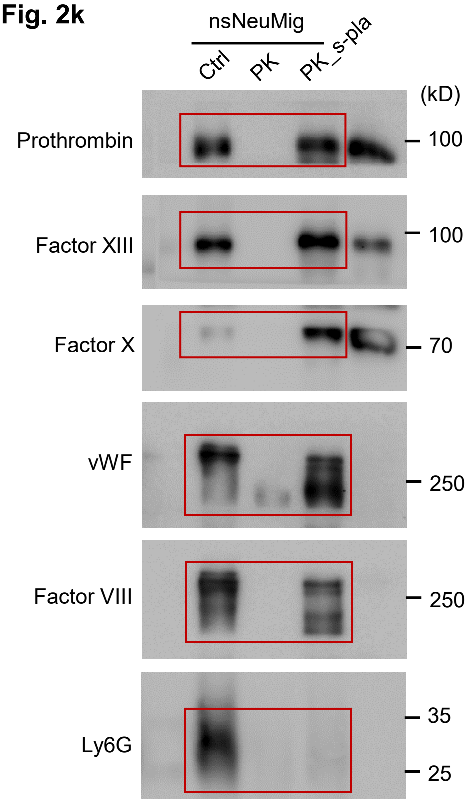

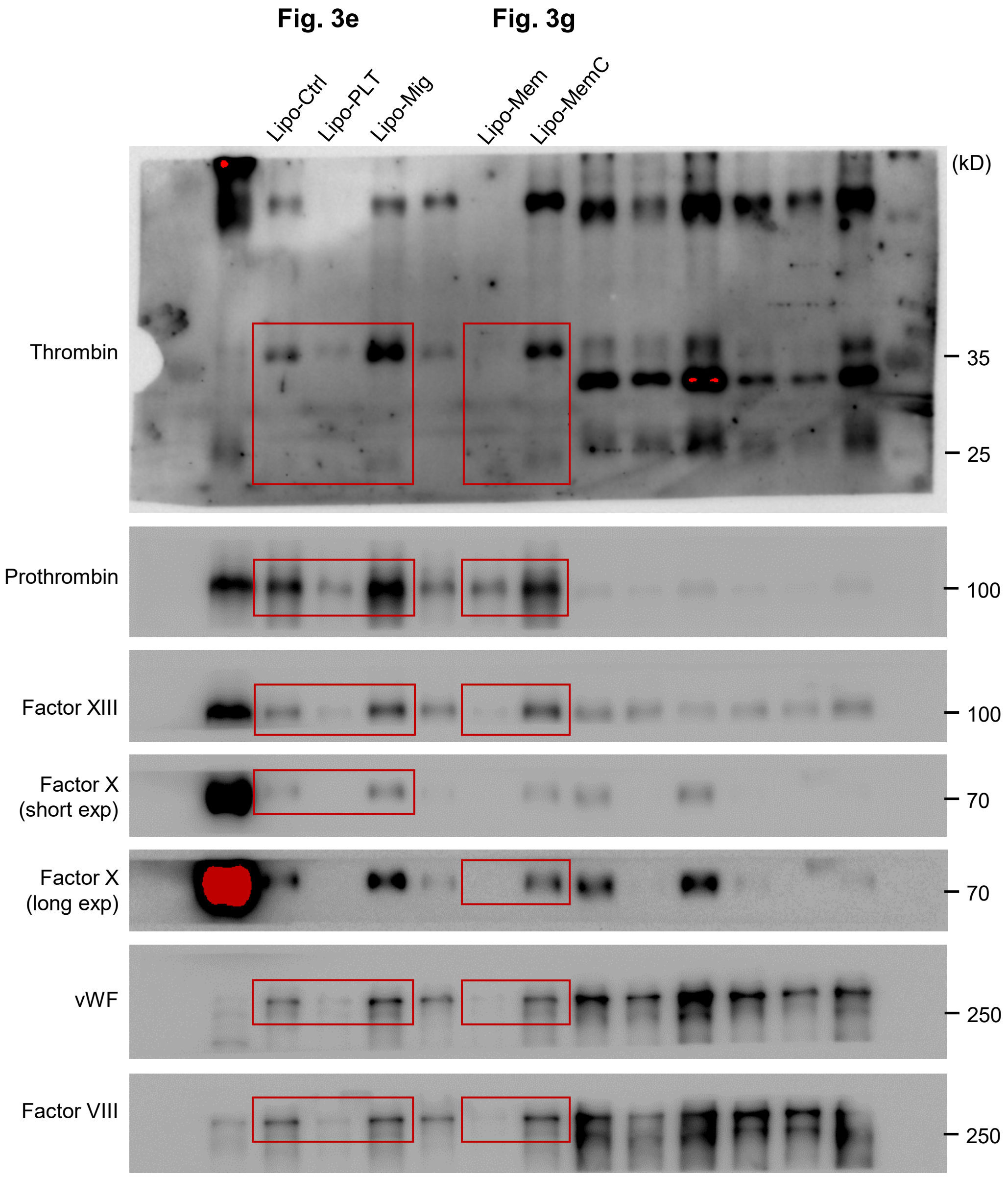

Fig. 3e
Fig. 3g
Lipo-Ctrl
Lipo-PLT
Lipo-Mig
Lipo-Mem
Lipo-MemC
(kD)
Thrombin
35
25
Prothrombin
100
Factor XIII
100
Factor X
(short exp)
70
Factor X
(long exp)
70
vWF
250
Factor VIII
250

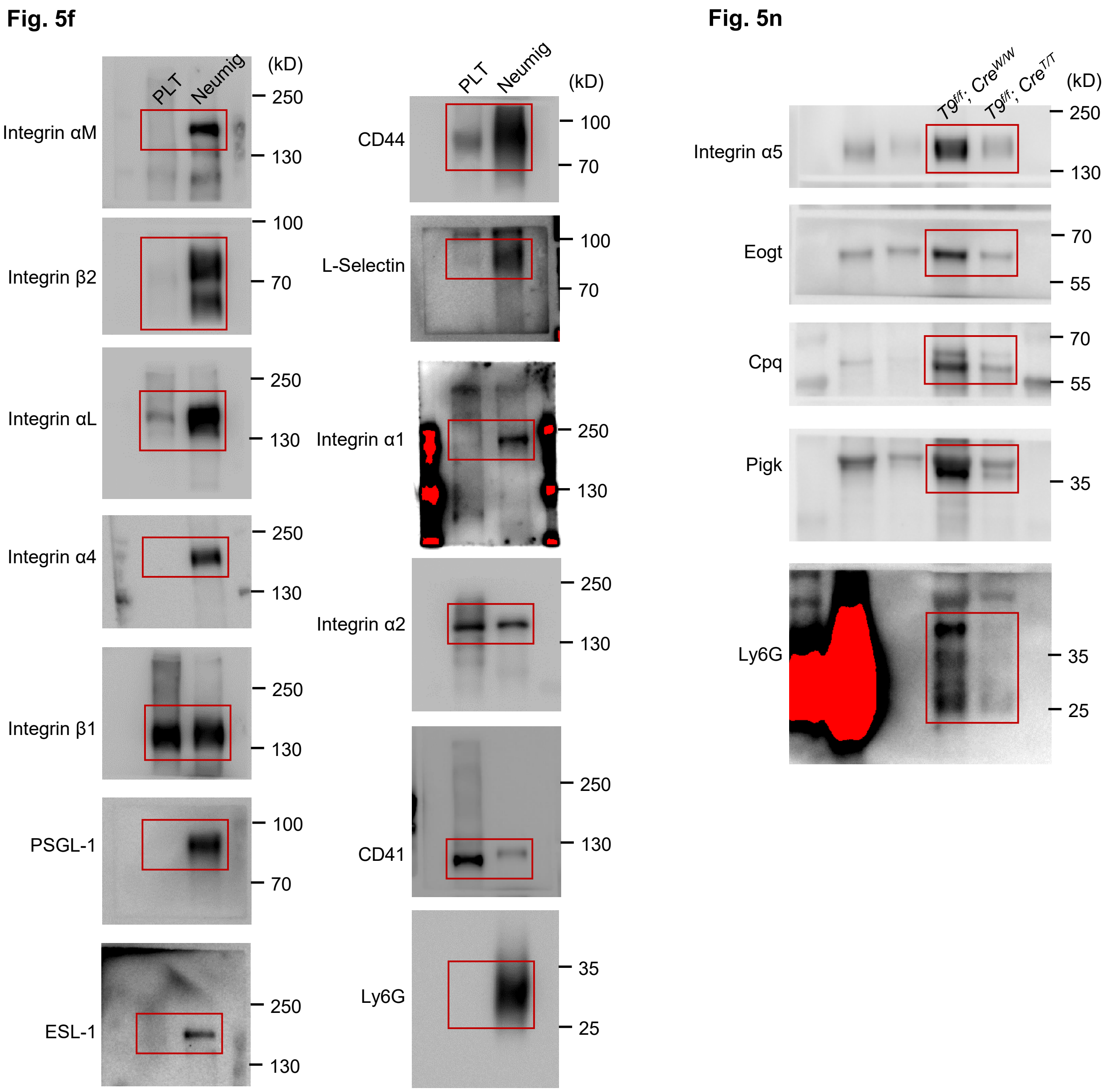
Fig. 5f
PLT
Neumig
(kD)
Integrin αM
250
130
Integrin β2
100
70
Integrin αL
250
130
Integrin α4
250
130
Integrin β1
250
130
PSGL-1
100
70
ESL-1
250
130
CD44
100
70
L-Selectin
100
70
Integrin α1
250
130
Integrin α2
250
130
CD41
250
130
Ly6G
35
25
Fig. 5n
T9f/f; Cre W/W
T9f/f; Cre T/T
Integrin α5
250
130
Eogt
70
55
Cpq
70
55
Pigk
35
Ly6G
35
25

Fig. 6g

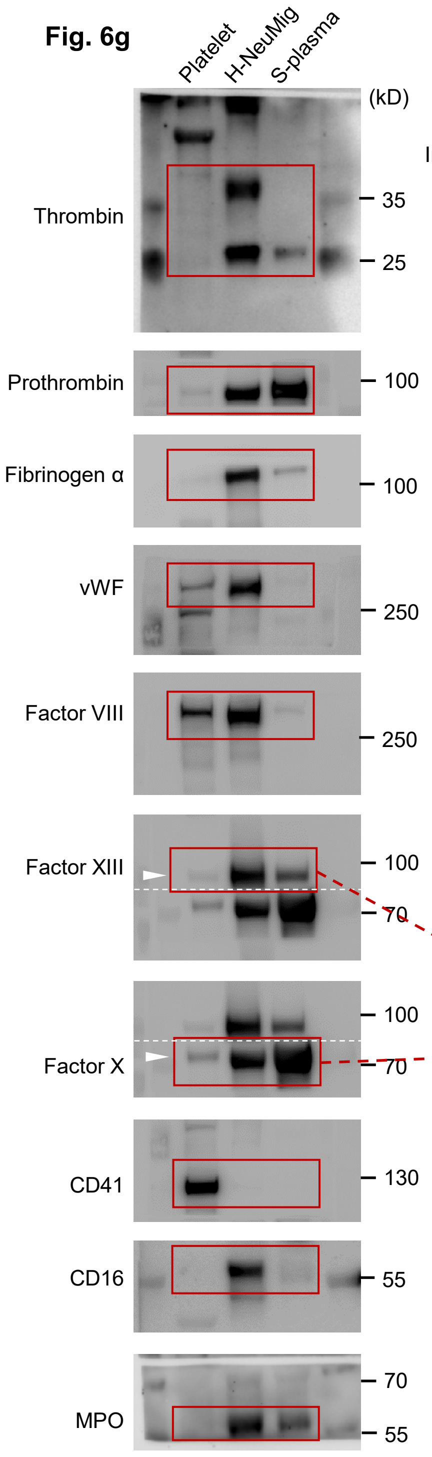

Fig. 7e

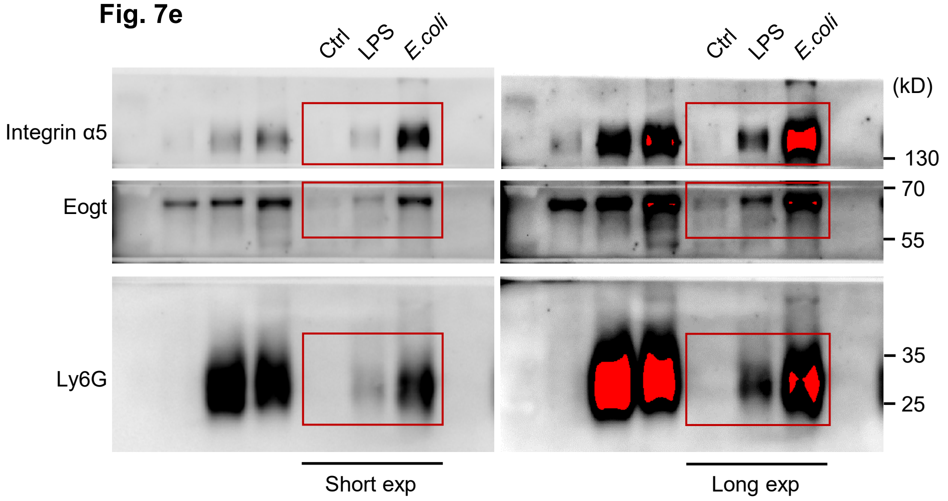

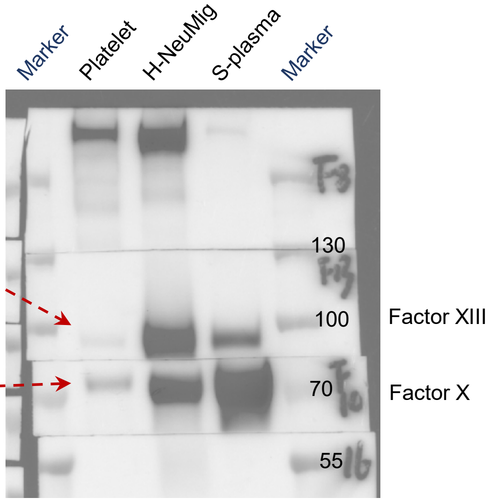

(Red rectangles correspond to cropped regions.
The dashed white lines indicate the cut off
boundary of the nitrocellulose membrane.
White arrowheads indicate the target bands)

Extended Data Fig. 1o

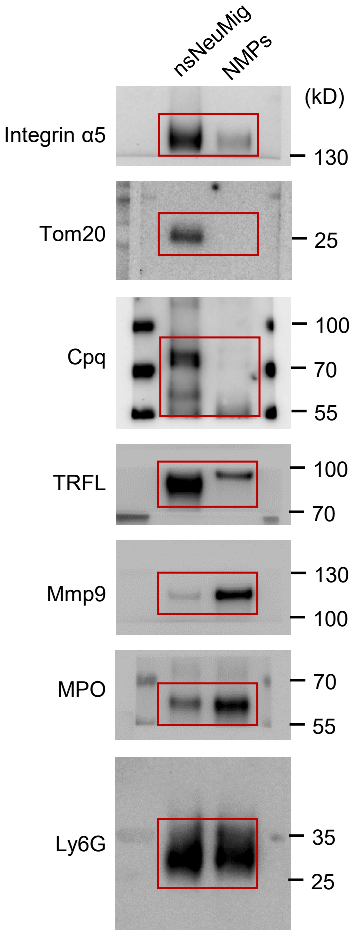

Extended Data Fig. 2a

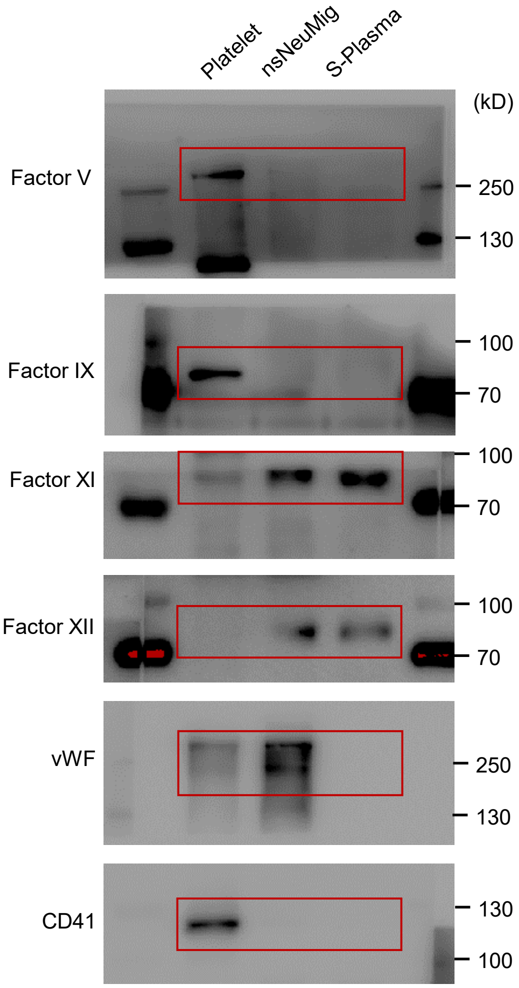

Extended Data Fig. 5e

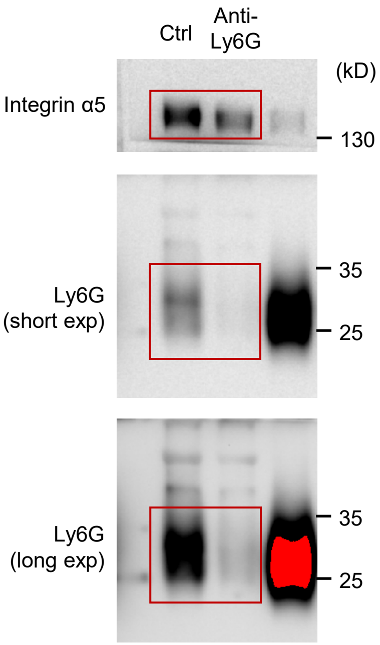

Extended Data Fig. 6a

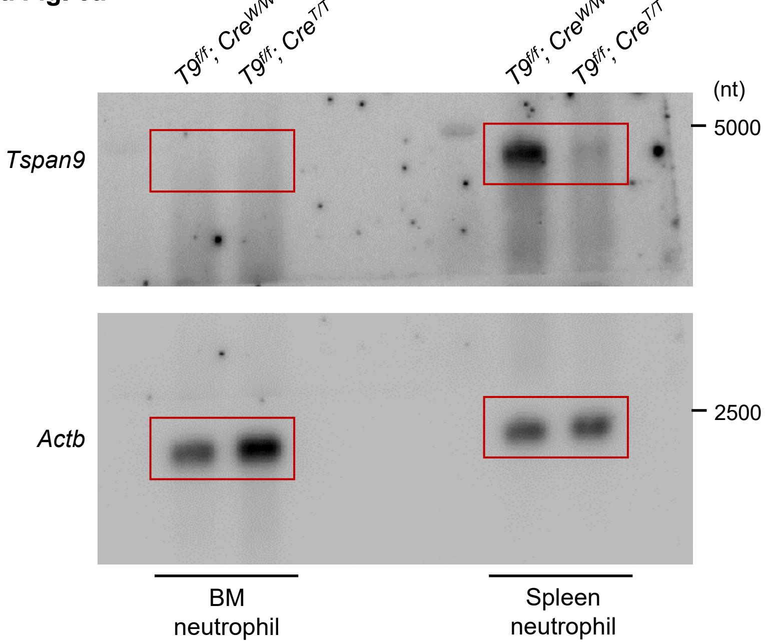

Extended Data Fig. 6b

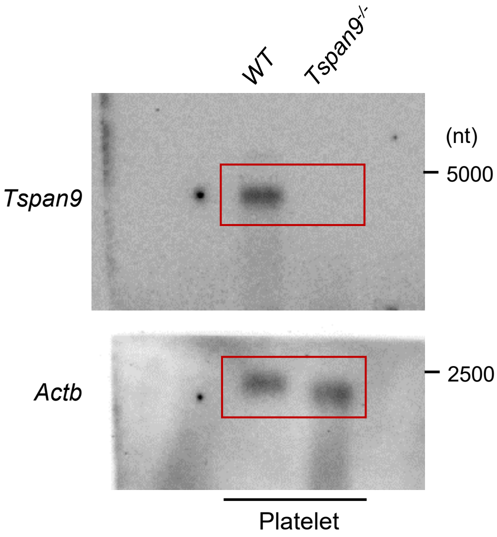

Extended Data Fig. 6c

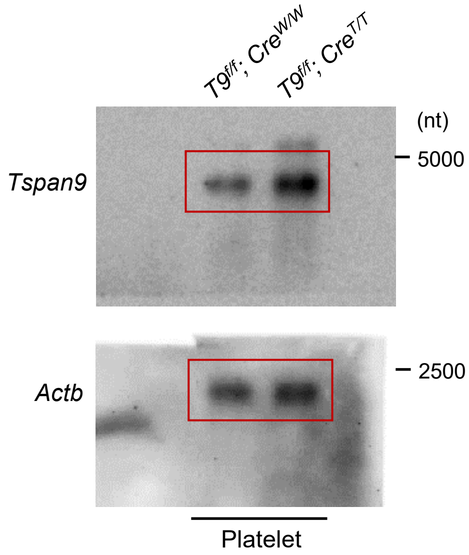

Extended Data Fig. 6i

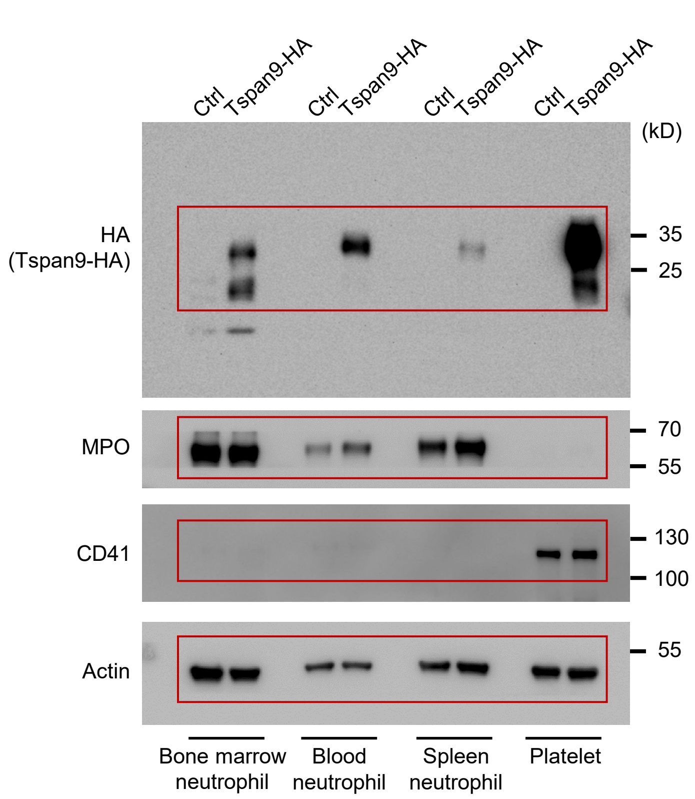

Extended Data Fig. 7b

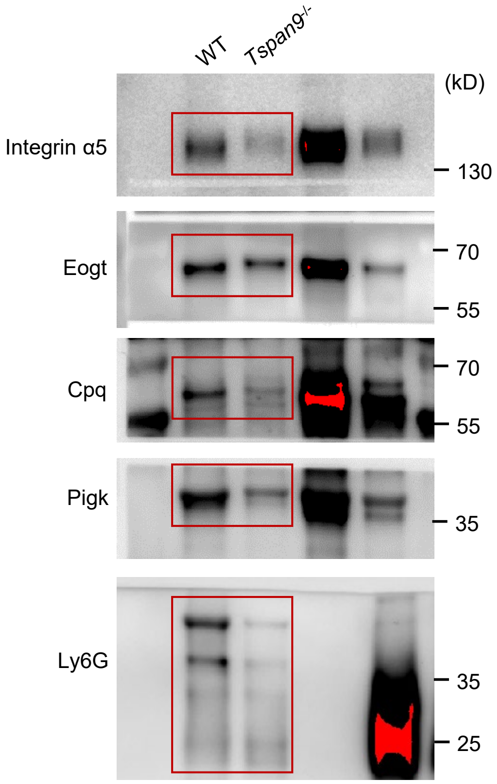

Alternative unprocessed and processed western blots

(Fig. 5n_EOGT and Extended Data Fig. 7b_Ly6G)

Replicate_uncropped_ Fig. 5n_EOGT

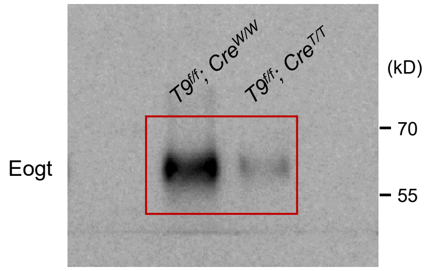

Replicate_cropped_ Fig. 5n_EOGT

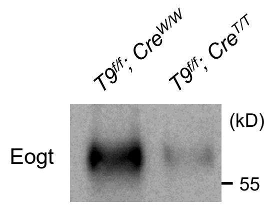

Replicate_uncropped_ED Fig. 7b_Ly6G

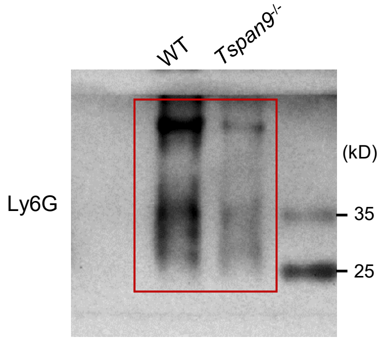

Replicate_cropped_ED Fig. 7b_Ly6G

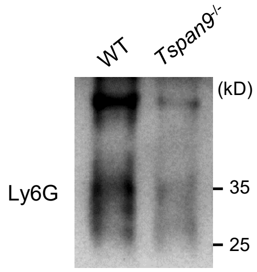

Supplement: Supplementary file 6 — A single file containing all of the unprocessed blots and/or gels for Figs. 1–7 and Extended Data Figs. 1–7. [file 41556_2024_1440_MOESM6_ESM.pdf]
